# Exploring entropy measures with topological indices on colorectal cancer drugs using curvilinear regression analysis and machine learning approaches

**DOI:** 10.1371/journal.pone.0327369

**Published:** 2025-07-09

**Authors:** Maria Fazal, Salma Kanwal, Muhammad Taskeen Raza, Asima Razzaque

**Affiliations:** 1 Department of Mathematics, Lahore College for Women University, Lahore, Pakistan; 2 Department of Electrical Engineering, Lahore College for Women University, Lahore, Pakistan; 3 Preparatory Year, Basic Science, King Faisal University, Al-Ahsa, Saudi Arabia; 4 Department of Mathematics, College of Science, King Faisal University, Saudi Arabia; Kwara State University, NIGERIA

## Abstract

A topological index is a numerical value derived from the structure of a molecule or graph that provides useful information about the molecule’s physical, chemical, or biological properties. These indices are especially important in chemo-informatics and QSAR/QSPR (Quantitative Structure-Activity Relationship/Quantitative Structure-Property Relationship) studies, where they are used to predict a wide range of properties without the need for experimental measurements. In essence, a topological index is a way to quantify the molecular structure in a form that can be used in mathematical models to estimate the molecule’s behavior, activity, or properties. In terms of chemical graph theory and chemo-informatics, entropy-based indices quantify the structural complexity or disorder in a molecule’s connectivity. These indices are useful for modeling and predicting molecular properties and biological activities. In this paper, we established a QSPR analysis of colorectal drugs between entropy indices and their physical properties and developed a relationship. Through a comprehensive analysis of these drugs, we gain essential insights into their molecular properties, which are vital for predicting their behavior and effectiveness in treating colorectal cancer. These models are compared with existing degree-based models, highlighting the superior performance of our approach. The QSPR study is performed using curvilinear regression models including linear, quadratic, cubic exponential and logarithmic models. Additionally, we propose the integration of machine learning (ML) techniques to further enhance the predictive accuracy and robustness of our models. By leveraging advanced ML algorithms, we aim to uncover more complex, non-linear relationships between topological indices and drug efficacy, potentially leading to more accurate predictions and better-informed drug design strategies.

## Introduction

Graph theory, a cornerstone of discrete mathematics, provides a versatile framework for modeling relationships and interactions across diverse fields such as computer science, biology, and social network analysis. By representing entities as vertices and their connections as edges, graph theory enables the study of structures like paths, cycles, and connectivity, offering insights into both theoretical and practical problems. Its applications range from optimizing transportation networks to understanding the spread of diseases in populations. As a discipline, graph theory not only advances mathematical understanding but also serves as a critical tool for addressing real-world challenges, making it indispensable in modern research and innovation [1]. Chemical Graph Theory and QSAR (Quantitative Structure-Activity Relationship)/QSPR (Quantitative Structure-Property Relationship) analysis are deeply intertwined, as both use molecular structures to predict and understand chemical properties and biological activities [2]. Cancer is a broad group of diseases that involves abnormal cell growth in the body. These abnormal cells can divide uncontrollably, form tumors, and potentially spread to other parts of the body through the blood and lymphatic systems, a process known as metastasis [3].

In a healthy body, cells grow, divide, and die in a controlled manner. However, in cancer, this process becomes disrupted. Cancer cells avoid the normal regulatory mechanisms that control cell division, leading to excessive growth and tumor formation.

Colorectal cancer is a type of cancer starts in the colon (large intestine) or the rectum, which are parts of the digestive system. It is one of the most common types of cancer worldwide, but with early detection and treatment, the chances of successful outcomes have improved significantly. There are two types of colorectal cancer, colon cancer and rectal cancer. Colon cancer starts in the colon, which is the long, tube-shaped structure that makes up the large intestine, and rectal cancer starts in the rectum, the last few inches of the large intestine, right before the anus. Most colorectal cancers begin as polyps, which are growths in the inner lining of the colon or rectum. Over time, some of these polyps can become cancerous. There are certain factors that increase the risk of developing colorectal cancer. The risk increases after age 50, although it can occur earlier. It also includes family history, processed meat diet (low in fiber, high in unhealthy fats), sedentary lifestyle, obesity, smoking, and the use of alcohol may increase risk. The symptoms of colorectal cancer can vary depending on the size and location of the tumor, as well as the stage of the cancer. Common symptoms of colorectal cancer include changes in bowel habits, blood in the stool, abdominal discomfort, persistent cramps, unexplained bloating, and anemia. The latest evidence on the epidemiology, risk factors, and prevention of colorectal cancer are discussed in [4], trends in colorectal cancer epidemiology and risk factors see [5]. Colorectal cancer is a major health concern, but early detection through screening, lifestyle changes, and effective treatments offer the potential for prevention and cure.

The 5-year survival rate for colorectal cancer varies significantly based on the stage at diagnosis. For localized cancer (Stage 0–I), the survival rate is 90% or higher, as early treatment, typically surgery, is very effective. For regional spread to nearby lymph nodes (Stage II–III), the survival rate is around 70–75%. However, for advanced cancer that has spread to distant organs (Stage IV), the 5-year survival rate drops to about 15–20%. Overall, the 5-year survival rate for colorectal cancer is approximately 64–65%. Early detection through regular screening is crucial, as it greatly improves survival rates, particularly in the early stages. Factors like age, overall health, and treatment response also influence outcomes [6].

Cancer drug discovery is particularly challenging due to the complexity of cancer biology, the heterogeneity of tumors, and the need for drugs that selectively target cancer cells without harming normal tissues. Tumors can develop resistance to treatments, and identifying compounds that are both effective and safe is a daunting task. QSPR (Quantitative Structure-Property Relationship) analysis plays a crucial role by using computational models to predict the biological activity, pharmacokinetics, and toxicity of potential cancer drugs based on their chemical structure. Through QSPR, researchers can screen large chemical libraries, optimize drug properties (like solubility, bioavailability, and specificity), and predict adverse effects early. It enhances drug discovery efficiency, reduces costs, and helps optimize drugs’ pharmacokinetic and pharmacodynamic profiles, leading to faster development timelines and higher chances of clinical success . Chemical graph theory is a branch of mathematical chemistry that applies graph theory to represent molecules and chemical structures. In this approach, atoms are represented as vertices (nodes), and bonds between atoms are represented as edges (links) of a graph. Chemical graph theory provides a way to model and analyze the structure of molecules using mathematical methods, offering insights into their chemical properties, reactivity, and behavior.

QSAR/QSPR analysis used to predict the biological activity and chemical properties of compounds using computational models that may be effective in treating colorectal cancer. By analyzing the relationship between molecular structure and cancer cell inhibition or therapeutic efficacy, QSAR models can help identify promising drug candidates that target specific molecular pathways involved in colorectal cancer [7]. For example, QSAR models might predict the anticancer activity of small molecules by correlating their chemical descriptors (such as molecular weight, and polar surface area) with their ability to inhibit kinase activity or disrupt DNA repair mechanisms in cancer cells. Similarly, QSPR models can be used to predict the physicochemical properties of potential drug candidates, such as solubility, polarizability, and toxicity, aiding in the design of drugs with optimal characteristics for colorectal cancer treatment. Graph entropy is a concept derived from information theory and applied to the structure of graphs (networks). It measures the complexity or randomness of a graph in terms of its topology. In Quantitative Structure-Activity Relationship (QSAR) or Quantitative Structure-Property Relationship (QSPR) analysis, the goal is to establish a mathematical model that correlates the chemical structure of molecules (represented as graphs) with their biological activity or physicochemical properties. Shannon entropy of a graph can be a useful descriptor for such analysis, particularly in drug design, because it captures key information about the complexity and diversity of the molecule’s structure, which can be linked to various chemical and biological properties [8].

Entropy indices are valuable in QSPR analysis of drugs because they capture the structural diversity and complexity of molecular graphs, which traditional indices may not fully represent. Traditional descriptors like molecular weight, topological indices, or logP focus on specific, often linear aspects of a molecule’s structure, but they may miss important features such as molecular flexibility or topological diversity, which are critical for biological activity [9]. Entropy indices, such as Shannon entropy, quantify the distribution and variability of atomic or bond arrangements, reflecting a molecule’s potential for diverse interactions with biological targets, which is often linked to drug efficacy, receptor binding, and metabolic stability [10]. Moreover, entropy measures help account for non-linear relationships between structure and activity, improving model robustness and predictive power in complex drug datasets [11]. Thus, entropy indices provide an additional layer of information that enhances QSPR models, particularly for novel drug candidates with complex structures. In information theory, entropy measures the uncertainty or unpredictability of information content. It was introduced by Claude Shannon in 1948 as a way to quantify the information contained in a message or data source [12].

Chu *et al*. [13], computed degree-based entropies using topological indices. The numerical and graphical representations of computed findings were merged with curve fitting of determined information entropies to topological indices. The underlying architecture is based on topological indices, which offer a comprehensive image of potentially essential traits that may be used in structural modification for graphite carbon nitride for certain applications.

Hui *et al*. [14], observed that the redefined third Zagreb entropy, Balaban entropy, and Randič entropy emerged as the most effective predictors. Specifically, Randič entropy and Balaban entropy demonstrated strong predictive capabilities for physiochemical properties, namely Henry’s Law and critical pressure, respectively. Additionally, the redefined third Zagreb entropy proved effective in predicting four other properties; enthalpy, molar mass, electronic energy, and molecular weight of benzene derivatives. Rauf *et al*. [15], examined that the redefined third Zagreb entropy is the most influential parameter, exhibiting strong predictive capability for the physical properties of boiling point and molecular weight of benzene derivatives. Arockiaraj *et al*. [16], created QSPR models for eighteen blood cancer therapeutic compounds by combining the reverse degree and entropy topological indices and compared the already given degree-based models, highlighting the higher efficacy of their method.

Zhang *et al*. [17], observed that there is a strong correlation between topological descriptors of some drugs structures and their physical properties in a number of tests every year. The considered chemical structures include hyaluronic acid’s paclitaxel conjugate, smart polymers, zig-zag polyomino chain, triangular benzenoid, and circumcoronene benzenoid. Nagesh and Siddiqui [18], computed the entropy measures for the complex structure of ruthenium bi-pyridine using Shannon’s entropy model. They presented a comparison between the Nirmala indices and their associated entropy measures through numerical computation. Further, the authors also determined a correlation between the Nirmala indices and associated entropy measurements using a regression model. The topological indices that we have used in order to calculate entropy indices are given in Table 1.

**Table 1:** Topological indices and their formulas.

Topological index	Notation	Formula
Atom Bond Connectivity Index [19]	ABC(G)	$\sum_{uv\in E(\text{G})}\sqrt{\frac{d_{u}+d_{v}-2}{d_{u}d_{v}}}$
Forgotten index [20]	F(G)	$\sum_{uv\in E(G)}({d_{u}}^{2}+{d_{v}}^{2})$
Geometric Arithmetic index [21]	GA(G)	$\sum_{uv\in E(\text{G})}\frac{2\sqrt{d_{u}d_{v}}}{d_{u}+d_{v}}$
The Harmonic index [22]	H(G)	$\sum_{uv\in E(\text{G})}\frac{2}{d_{u}+d_{v}}$
The inverse sum indeg index [23]	ISI(G)	$\sum_{uv\in E(\text{G})}\frac{d_{u}d_{v}}{d_{u}+d_{v}}$
The first Zagreb index [24]	${\text{M}}_{1}(\text{G})$	$\sum_{uv\in E(\text{G})}\left(d_{u}+d_{v}\right)$
The second Zagreb index [24]	${\text{M}}_{2}(\text{G})$	$\sum_{uv\in E(\text{G})}d_{u}d_{v}$
The sum connectivity index [25]	S(G)	$\sum_{uv\in E(\text{G})}\sqrt{\frac{1}{d_{u}+d_{v}}}$
The Sombor index [26]	SO(G)	$\sum_{uv\in E(\text{G})}\sqrt{{d_{u}}^{2}+{d_{v}}^{2}}$
The Randič index [27]	R(G)	$\sum_{uv\in E(\text{G})}\sqrt{\frac{1}{d_{u}d_{v}}}$

In the case of chemical networks pertaining to the topological index function, the entropy function is defined as

$$Ent_{\phi }(\text{G})=-\sum_{u^{\prime }v^{\prime }\in E(\text{G})}\frac{\phi (u^{\prime }v^{\prime })}{\sum_{uv\in E(\text{G})}}log\frac{\phi (u^{\prime }v^{\prime })}{\sum_{uv\in E(\text{G})}}$$ (1)

Now by using formulas given in the table and equation 1 we have the following entropy indices:

- Atom bond connectivity entropy
  $$Ent_{ABC}(\text{G})=log(ABC)-\frac{1}{\left(ABC\right)}\sum_{i=1}^{n}\sum_{uv\in E(\text{G})}log[[\sqrt{\frac{d_{u}+d_{v}-2}{d_{u}d_{v}}}{]}^{\left[\sqrt{\frac{d_{u}+d_{v}-2}{d_{u}d_{v}}}\right]}]$$ (2)
- Forgotten entropy
  $$Ent_{F}(\text{G})=log(F)-\frac{1}{\left(F\right)}\sum_{i=1}^{n}\sum_{uv\in E(\text{G})}log[({d_{u}}^{2}+{d_{v}}^{2}{)}^{\left({d_{u}}^{2}+{d_{v}}^{2}\right)}]$$ (3)
- Geometric Arithmetic entropy
  $$Ent_{GA}(\text{G})=log(GA)-\frac{1}{\left(GA\right)}\sum_{i=1}^{n}\sum_{uv\in E(\text{G})}log[[\frac{2\sqrt{d_{u}d_{v}}}{d_{u}+d_{v}}{]}^{\left[\frac{2\sqrt{d_{u}d_{v}}}{d_{u}+d_{v}}\right]}]$$ (4)
- Harmonic entropy
  $$Ent_{H}(\text{G})=log(H)-\frac{1}{\left(H\right)}\sum_{i=1}^{n}\sum_{uv\in E(\text{G})}log[[\frac{2}{d_{u}+d_{v}}{]}^{\left[\frac{2}{d_{u}+d_{v}}\right]}]$$ (5)
- Inverse sum indeg entropy
  $$Ent_{ISI}(\text{G})=log(ISI)-\frac{1}{\left(ISI\right)}\sum_{i=1}^{n}\sum_{uv\in E(\text{G})}log[[\frac{d_{u}d_{v}}{d_{u}+d_{v}}{]}^{\left(\frac{d_{u}d_{v}}{d_{u}+d_{v}}\right)}]$$ (6)
- Sum connectivity entropy
  $$Ent_{S}(\text{G})=log(S)-\frac{1}{\left(S\right)}\sum_{i=1}^{n}\sum_{uv\in E(\text{G})}log[[\sqrt{\frac{1}{d_{u}+d_{v}}}{]}^{\left[\sqrt{\frac{1}{d_{u}+d_{v}}}\right]}]$$ (7)
- Sombor entropy
  $$Ent_{SO}(\text{G})=log(SO)-\frac{1}{\left(SO\right)}\sum_{i=1}^{n}\sum_{uv\in E(\text{G})}log[(\sqrt{{d_{u}}^{2}+{d_{v}}^{2}}{)}^{(\sqrt{{d_{u}}^{2}+{d_{v}}^{2})}}]$$ (8)
- Randič entropy
  $$Ent_{R}(\text{G})=log(R)-\frac{1}{\left(R\right)}\sum_{i=1}^{n}\sum_{uv\in E(\text{G})}log[[\sqrt{\frac{1}{d_{u}d_{v}}}{]}^{\left[\sqrt{\frac{1}{d_{u}d_{v}}}\right]}]$$ (9)
- First Zagreb entropy
  $$Ent_{M_{1}}(\text{G})=log(M_{1})-\frac{1}{\left(M_{1}\right)}\sum_{i=1}^{n}\sum_{uv\in E(\text{G})}log[(d_{u}+d_{v}{)}^{\left(d_{u}+d_{v}\right)}]$$ (10)
- Second Zagreb entropy
  $$Ent_{M_{2}}(\text{G})=log(M_{2})-\frac{1}{\left(M_{2}\right)}\sum_{i=1}^{n}\sum_{uv\in E(\text{G})}log[(d_{u}d_{v}{)}^{\left(d_{u}d_{v}\right)}]$$ (11)

## Colorectal cancer drugs

**Fig 1:**
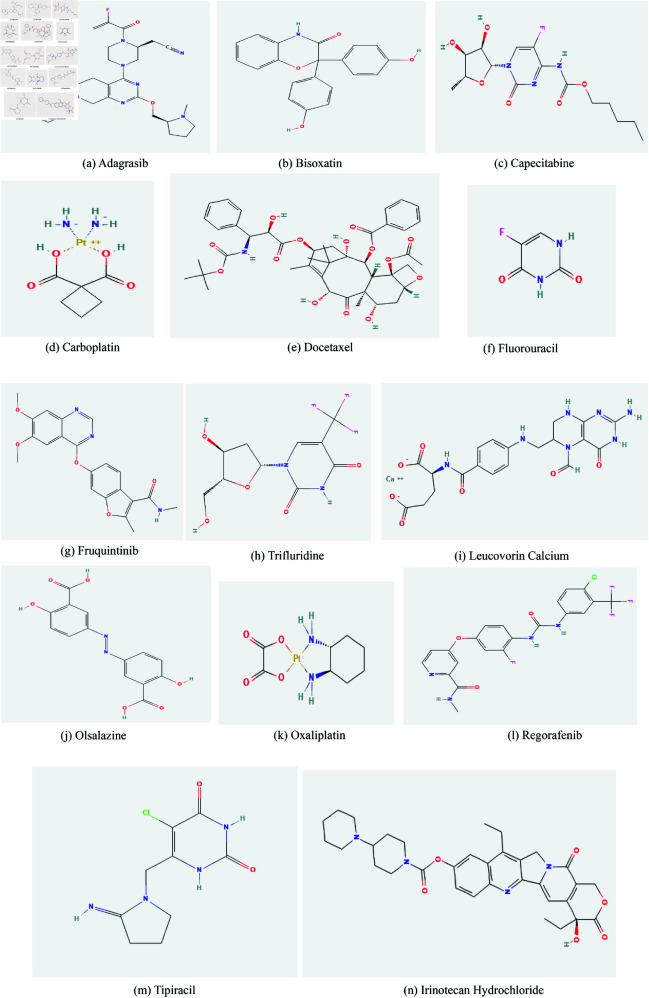
Molecular structures of anti-colorectal cancer drugs.

In our analysis, we consider 14 colorectal cancer drugs Adagrasib, Bisoxatin, Capecitabine, Carboplatin, Docetaxel, Fluorouracil, Fruquintinib Irinotecan Hydrochloride, Leucovorin Calcium, Olsalazine, Oxaliplatin, Regorafenib, Tiparicil, Triflouridine. The considered drugs are taken from ChemSpider and PubChem, chemical structure database, are shown in Fig 1. While performing calculations, molecular structures of these drugs are considered with depleted hydrogen atoms, in which atoms are considered as vertices and bonds between them as edges between the vertices of the graph. The degree *d*_*u*_ is the number of all the edges incident to it.

**Computation of entropy indices**

Let ${\text{G}}_{1}$ be a graph for Oxaliplatin with $|V({\text{G}}_{1})|=15$ and $|E({\text{G}}_{1})|=17$ and $ABC({\text{G}}_{1})=12.1587$, $F({\text{G}}_{1})=238$, $GA({\text{G}}_{1})=16.3826$. For this we need edge partition. If $E_{i,j}=\{uv\in ({\text{G}}_{1})|d_{u}=i,d_{v}=j\}$, where *d*_*u*_ denotes the degree of a vertex *u*, then for ${\text{G}}_{1}$ , we have,

$|E_{1,3}|=2$, $|E_{2,3}|=6$, $|E_{2,2}|=3$, $|E_{2,4}|=4$, $|E_{3,3}|=2$

By using numerical formulas of entropy indices mentioned above we have,


$${\mathrm{Ent}}_{F}(G_{1})=\log(238)-\frac{1}{\left(238\right)}[20\log(10)+36\log(18)+78\log(13)+80\log(20)+24\log8]=2.7843$$

$${\mathrm{Ent}}_{ABC}(G_{1})=\log(12.1587)-\frac{1}{\left(12.1587\right)}[2\sqrt{\frac{2}{3}}\log\sqrt{\frac{2}{3}}+2\sqrt{\frac{4}{9}}\log\sqrt{\frac{4}{9}}+6\sqrt{\frac{3}{6}}\log\sqrt{\frac{3}{6}}+4\sqrt{\frac{4}{8}}\log\sqrt{\frac{4}{8}}+3\sqrt{\frac{2}{4}}\log\sqrt{\frac{2}{4}}]=2.8317$$

$${\mathrm{Ent}}_{GA}(G_{1})=\log(16.3826)-\frac{1}{\left(16.3826\right)}[\frac{4\sqrt{3}}{4}\log\frac{2\sqrt{3}}{4}+\frac{4\sqrt{9}}{6}\log\frac{2\sqrt{9}}{6}+\frac{12\sqrt{6}}{5}\log\frac{2\sqrt{6}}{5}+\frac{8\sqrt{8}}{6}\log\frac{2\sqrt{8}}{6}+\frac{6\sqrt{4}}{4}\log\frac{2\sqrt{4}}{4}]=2.8323$$

The entropy indices for the other drugs considered with depleted hydrogen atoms are computed in the similar way and are given in the Table 2.

**Table 2:** Calculated values for entropy indices for the drugs.

Name of drugs	Ent_*ABC*_	Ent_*F*_	Ent_*GA*_	Ent_*H*_	Ent_*ISI*_	${\mathrm{Ent}}_{M_{1}}$	${\mathrm{Ent}}_{M_{2}}$	Ent_*S*_	Ent_*SO*_	Ent_*R*_
Adagrasib	3.8696	3.8292	3.87	3.8571	3.8504	3.8582	3.81	3.8677	3.8601	3.8528
Bisoxatin	3.3305	3.2658	3.3315	3.3177	3.2823	3.3154	3.253	3.3284	3.3163	3.3147
Capecitabine	3.2554	3.2124	3.256	3.2412	3.1868	3.242	3.1729	3.2539	3.246	3.2337
Carboplatin	2.635	2.4352	3.3295	2.6199	2.602	2.62	2.539	2.634	2.622	2.614
Docetaxel	4.1384	4.0772	4.1404	4.1251	4.1041	4.1237	4.0354	4.1385	4.1264	4.1177
Fluorouracil	2.194	2.1717	2.888	2.188	2.169	2.187	2.1284	2.1948	2.1909	2.1795
Fruquintinib	3.4646	3.4192	3.4651	3.44501	3.4353	3.4501	3.4002	3.4609	3.4523	3.4401
IrinotecanHCl	3.8901	3.8369	3.8909	3.8733	3.868	3.8757	3.8212	3.8874	3.8772	3.8695
LeucovorinCa	3.5813	3.5476	3.5822	3.57	3.5582	3.5711	3.5164	3.5801	3.5634	3.5634
Olsalazine	3.1328	3.1049	3.1338	3.1247	3.1082	3.1242	3.0663	3.1327	3.1278	3.1168
Oxaliplatin	2.8317	2.7843	2.8323	2.8198	2.816	2.82	2.7807	2.8299	2.8204	2.8169
Ragorafenib	3.5523	3.5161	3.5532	3.543	3.5316	3.5442	3.4946	3.5523	3.5451	3.4903
Tipricil	2.8306	2.8043	2.8317	2.823	2.8077	2.8226	2.7686	2.8306	2.8259	2.8151
Trifluridine	3.0391	3.0028	3.0416	3.0315	3.0084	3.0302	2.9596	3.0404	3.033	3.019

## Quantitative-structure property relationship (QSPR analysis)

Regression models are statistical techniques used to understand relationships between variables and to predict the value of a dependent (target) variable based on one or more independent (predictor) variables. In the context of QSPR (Quantitative Structure-Property Relationship) and QSAR (Quantitative Structure-Activity Relationship) analysis, regression models are employed to correlate molecular descriptors (independent variables) with a chemical property or biological activity (dependent variable). In this section, we investigate the QSPR analysis of the entropy indices and physical properties of colorectal cancer drugs. The physio-chemical properties that we have considered are Molecular Weight (MW), Topological Surface Area (TSA), Complexity (C), Density (D), Molar Volume (MV), Polarizability (P) and Molar Refractivity (MR). The data set for the mentioned physio-chemical properties are taken from PubChem and DrugBank, databases used for drug discovery, given in the Table 3.

**Table 3:** Physical properties for the drugs.

Name of drugs	MW	TSA	Complexity	Density	MV	Polarizability	MR
Adagrasib	604.1	88.8	1060	1.3	466.2	64.8	166.79
Bisoxatin	333.3	78.8	458	1.4	243.5	36.3	91.5
Capecitabine	359.35	121	582	1.59	240.5	32.6	82.3
Carboplatin	371.25	82.3	153	1.7	218	18.27	60.04
Docetaxel	807.9	224	1660	1.4	585.7	81.4	205.2
Fluorouracil	130.08	58.2	199	1.5	84.6	9.46	26.17
Fruquintinib	393.4	95.7	579	1.3	302.1	43.1	108.7
Irinotecan HCl	623.1	113	1200	1.4	416.8	63.1	159.1
Leucovorin Ca	511.5	221	900	1.81	281	46.33	126.66
Olsalazine	302.24	140	415	1.6	194.1	29	73.2
Oxaliplatin	397.3	132	124	1.5	265	21.9	67.52
Ragorafenib	482.8	92.4	686	1.5	323.7	44.8	113.1
Tipricil	242.66	85.3	404	1.7	141.4	22.7	57.2
Trifluridine	296.2	99.1	464	1.6	179.9	22.2	55.9

**Fig 2:**
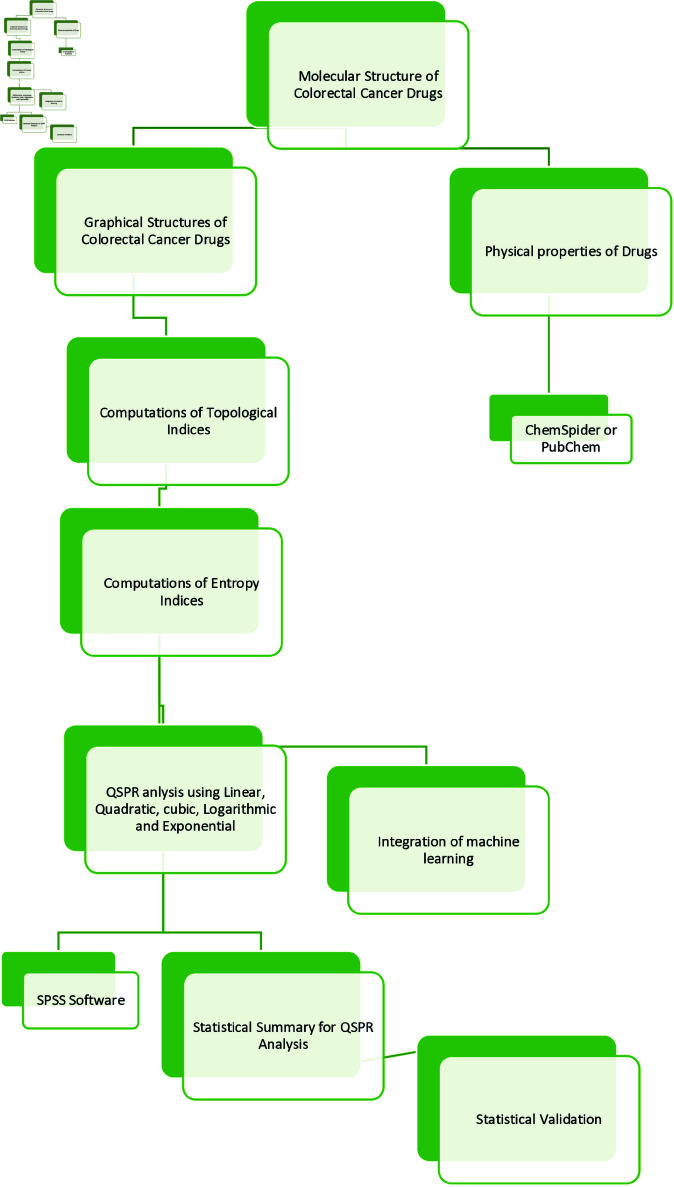
Flowchart for regression analysis using entropy measure.

The whole procedure is shown in Fig 2. We analyze the entropy indices with physical properties using following regression models:

*Y* = *a* + *bX* (linear equation)

$Y=a+b_{1}X+b_{2}X^{2}$ (quadratic equation)

$Y=a+b_{1}X+b_{2}X^{2}+b_{3}X^{3}$ (cubic equation)

Y=a+b.lnX (logarithmic equation)

*Y* = *a*.*b*^*X*^ (exponential equation)

where *Y* is dependent variable, *X* is independent variable, *a* is the intercept value and *b*_*i*_’s represent the strength and direction of the relationship between independent and dependent variable.

Using above mentioned equations we obtain the following regression models (best-fitted) for the entropy indices:

$$\text{Molecular Weight}=323.0+18.4\;({\text{Ent}}_{ISI})-75.9\;({\text{Ent}}_{ISI}{)}^{2}+24.7\;({\text{Ent}}_{ISI}{)}^{3}$$

$$\text{Complexity}=756.7+4.4\;({\text{Ent}}_{F})-309.8\;({\text{Ent}}_{F}{)}^{2}+88.3\;({\text{Ent}}_{F}{)}^{3}$$

$$\text{Complexity}=724.6+2.1\;({\text{Ent}}_{R})-295.3\;({\text{Ent}}_{R}{)}^{2}+84.4\;({\text{Ent}}_{R}{)}^{3}$$

$$\text{Molar Volume}=257+15.6\;({\text{Ent}}_{SO})-73\;({\text{Ent}}_{SO}{)}^{2}+22.1\;({\text{Ent}}_{SO}{)}^{3}$$

$$\text{Polarizability}=2.3-6.2\;({\text{Ent}}_{ABC})+2.4\;({\text{Ent}}_{ABC}{)}^{2}+15.2\;({\text{Ent}}_{ABC}{)}^{3}$$

$$\text{Polarizability}=13.8+1.7\;({\text{Ent}}_{R})-5.8\;({\text{Ent}}_{R}{)}^{2}+2.4\;({\text{Ent}}_{R}{)}^{3}$$

$$\text{Molar Refractivity}=47.3+4.6({\text{Ent}}_{ISI})-16.4({\text{Ent}}_{ISI}{)}^{2}+6.3({\text{Ent}}_{ISI}{)}^{3}$$

$$\text{Molar Refractivity}=46.6+3.7({\text{Ent}}_{R})-16.3({\text{Ent}}_{R}{)}^{2}+6.2({\text{Ent}}_{R}{)}^{3}$$

## Computations of statistical parameters

In order to analyze the physical properties of colorectal cancer, the computed entropy indices and the considered properties are given in section 1. For this purpose we compute the statistical parameters intercept value (*a*), regression coefficients (*b*_*i*_’s), correlation coefficient (*r*), coefficient of determination (${\text{r}}^{2}$), Fisher’s constant (*F*), standard error of estimate (*SE*) and *p*-value. The regression analysis presented in Table 4 demonstrates that the entropy-based molecular descriptor *Entr*_*ABC*_ is a strong and statistically significant predictor of the target property. The unstandardized coefficient for *Entr*_*ABC*_ is 290.601, indicating that each unit increase in this descriptor results in an approximate 290.6 unit increase in the response variable. The standardized beta coefficient of 0.891 reflects a strong positive relationship, and the associated p-value (0.000) confirms its high statistical significance. Additionally, the 95% confidence interval for this coefficient (197.417, 383.786) does not include zero, further supporting the reliability of the model. The intercept of the model is –531.321, with a p-value of 0.003, showing that the model is well-calibrated even in the absence of the predictor. The statistical parameters are given in the Tables 5, 6, 7, 8, 9 and 10. The correlation coefficient (r) measures the strength and direction of the linear relationship between two variables, ranging from -1 (perfect negative correlation) to  + 1 (perfect positive correlation). In contrast, the coefficient of determination ${\text{r}}^{2}$ represents the proportion of variance in the dependent variable (*Y*) that is explained by the independent variable (*X*) in the regression model, ranging from 0 to 1. A high F-value indicates that the model is statistically significant.The p-value represents the probability of obtaining a test statistic at least as extreme as the one observed, assuming the null hypothesis *H*_0_ is true. p-value ≤0.05: Reject the null hypothesis; the result is statistically significant. p-value >0.05, fail to reject the null hypothesis; insufficient evidence to conclude a significant effect. The standard error (SE) in a regression model is a measure of the variability or uncertainty in estimates. A smaller standard error indicates a more precise estimate of the coefficient and a large value of standard error suggest greater variability in the coefficient estimate, making it less reliable. The correlation coefficient for all the curvilinear regression models are given in the Table 11 and coefficient of determination are given in the Table 12. From our computations we have the following results:

**Table 4:** Confidence interval for MW versus *Ent*_*ABC*_.

Model	Unstandardized Coefficients		Standardized Coefficients	Sig.	95.0%Confidence Interval	
	B	Std. Error	Beta		Lower Bound	Upper Bound
(Constant)	-531.321	141.483		0.003	-839.585	-223.057
*Entr*_*ABC*_	290.601	42.769	0.891	0.000	197.417	383.786

**Table 5:** Statistical parameters for the Molecular Weight.

Entropy Index	$b_{1}$	$b_{2}$	$b_{3}$	a	r	$r^{2}$	SE	F	p-value	Indicator
**Exponential**										
Ent_*ABC*_	0.8	-	-	32.1	0.9	0.81	0.2	50.5	0.000	Significant
Ent_*F*_	0.7	-	-	37.4	0.87	0.76	0.2	37.9	0.000	Significant
Ent_*GA*_	0.8	-	-	31.9	0.9	0.81	0.2	50.6	0.000	Significant
Ent_*H*_	0.8	-	-	32.2	0.9	0.81	0.2	49.9	0.000	Significant
Ent_*ISI*_	0.8	-	-	32.7	0.9	0.81	0.2	51.6	0.000	Significant
${\mathrm{Ent}}_{M_{1}}$	0.8	-	-	32.3	0.9	0.81	0.2	50	0.000	Significant
${\mathrm{Ent}}_{M_{2}}$	0.8	-	-	33.9	0.9	0.8	0.2	49	0.000	Significant
Ent_*S*_	0.8	-	-	32.1	0.9	0.81	0.2	50.4	0.000	Significant
Ent_*SO*_	0.8	-	-	32.2	0.9	0.81	0.2	49.6	0.000	Significant
Ent_*R*_	0.8	-	-	32.3	0.9	0.81	0.2	50.1	0.000	Significant
**Linear**										
Ent_*ABC*_	290.6	-	-	-531.3	0.89	0.79	82.7	46.2	0.000	Significant
Ent_*F*_	278.6	-	-	-477.6	0.87	0.75	90.2	36.9	0.0001	Significant
Ent_*GA*_	291.4	-	-	-534.4	0.89	0.79	82.4	46.5	0.000	Significant
Ent_*H*_	291.5	-	-	-530.6	0.89	0.79	82.8	45.9	0.000	Significant
Ent_*ISI*_	291.6	-	-	-525.9	0.89	0.8	81.6	47.7	0.000	Significant
${\mathrm{Ent}}_{M_{1}}$	291.1	-	-	-529.6	0.89	0.79	82.9	45.8	0.000	Significant
${\mathrm{Ent}}_{M_{2}}$	290.1	-	-	-509	0.89	0.79	84.3	44	0.000	Significant
Ent_*S*_	290.9	-	-	-532.1	0.89	0.79	82.6	46.2	0.000	Significant
Ent_*SO*_	291.4	-	-	-531.2	0.89	0.79	83	45.7	0.000	Significant
Ent_*R*_	292.3	-	-	-530.6	0.79	0.78	82.5	46.4	0.000	Significant
**Quadratic**										
Ent_*ABC*_	-641.9	145.6	-	922.2	0.93	0.87	69.3	35.9	0.000	Significant
Ent_*F*_	-822.6	175.4	-	1201.7	0.93	0.86	71.7	33.1	0.000	Significant
Ent_*GA*_	-642.8	145.7	-	923.6	0.93	0.87	69.1	36.1	0.000	Significant
Ent_*H*_	-650.9	147.7	-	933.3	0.93	0.87	69.2	36	0.000	Significant
Ent_*ISI*_	-618.8	143.2	-	881.7	0.93	0.87	68.6	36.7	0.000	Significant
${\mathrm{Ent}}_{M_{1}}$	-651	147.6	-	933.5	0.93	0.87	69.3	35.9	0.000	Significant
${\mathrm{Ent}}_{M_{2}}$	-665.2	152.9	-	942.1	0.93	0.86	70.5	34.4	0.000	Significant
Ent_*S*_	-643.5	145.9	-	924.4	0.93	0.87	69.2	36	0.000	Significant
Ent_*SO*_	-651.1	147.6	-	934.1	0.93	0.87	69.5	35.7	0.000	Significant
Ent_*R*_	-627.1	144.4	-	894.6	0.87	0.84	69.5	35.7	0.000	Significant
**Cubic**										
Ent_*ABC*_	18.4	-75.9	24.7	323	0.94	0.88	66.5	39.4	0.000	Significant
Ent_*F*_	19.1	-105.7	31.2	422	0.93	0.87	68.8	36.5	0.000	Significant
Ent_*GA*_	18.3	-75.7	24.7	322.3	0.94	0.88	66.4	39.5	0.000	Significant
Ent_*H*_	18.3	-77.3	25.1	326.3	0.94	0.88	66.5	39.4	0.000	Significant
Ent_*ISI*_	18.6	-73	24.4	312.2	0.94	0.88	65.9	40.2	0.000	Significant
${\mathrm{Ent}}_{M_{1}}$	18.5	-77.6	25.2	327.4	0.94	0.88	66.6	39.3	0.000	Significant
${\mathrm{Ent}}_{M_{2}}$	20.2	-83.5	27.1	340.2	0.93	0.87	67.6	38	0.000	Significant
Ent_*S*_	18.2	-76	24.7	323	0.94	0.88	66.4	39.5	0.000	Significant
Ent_*SO*_	18.4	-77.3	25.1	326.4	0.94	0.88	66.8	39.1	0.000	Significant
Ent_*R*_	17.9	-73	24.4	311.9	0.88	0.85	66.9	38.9	0.000	Significant

**Table 6:** Statistical parameters for the Complexity.

Entropy Index	$b_{1}$	$b_{2}$	$b_{3}$	a	r	$r^{2}$	SE	F	p-value	Indicator
**Exponential**										
Ent_*ABC*_	1.3	-	-	7.4	0.91	0.82	0.3	56.1	0.000	Significant
Ent_*F*_	1.3	-	-	8	0.92	0.85	0.3	65.9	0.000	Significant
Ent_*GA*_	1.3	-	-	7.3	0.91	0.82	0.3	56.3	0.000	Significant
Ent_*H*_	1.3	-	-	7.3	0.91	0.83	0.3	57	0.000	Significant
Ent_*ISI*_	1.3	-	-	7.7	0.91	0.82	0.3	54.8	0.000	Significant
${\mathrm{Ent}}_{M_{1}}$	1.3	-	-	7.4	0.91	0.83	0.3	56.8	0.000	Significant
${\mathrm{Ent}}_{M_{2}}$	1.3	-	-	8	0.91	0.82	0.3	54.9	0.000	Significant
Ent_*S*_	1.3	-	-	7.3	0.91	0.82	0.3	56.5	0.000	Significant
Ent_*SO*_	1.3	-	-	7.3	0.91	0.83	0.3	57.1	0.000	Significant
Ent_*R*_	1.3	-	-	7.4	0.91	0.82	0.3	56.2	0.000	Significant
**Linear**										
Ent_*ABC*_	734.1	-	-	-1764.1	0.9	0.81	196.3	52.2	0.000	Significant
Ent_*F*_	721.5	-	-	-1684.9	0.9	0.81	196.9	51.8	0.000	Significant
Ent_*GA*_	736.3	-	-	-1772.4	0.9	0.81	195.6	52.7	0.000	Significant
Ent_*H*_	737.3	-	-	-1765.8	0.9	0.81	195.6	52.7	0.000	Significant
Ent_*ISI*_	734.6	-	-	-1743.7	0.9	0.81	195.8	52.5	0.000	Significant
${\mathrm{Ent}}_{M_{1}}$	736.1	-	-	-1762.4	0.9	0.81	196	52.4	0.000	Significant
${\mathrm{Ent}}_{M_{2}}$	732.4	-	-	-1706.3	0.9	0.8	201.2	49.2	0.000	Significant
Ent_*S*_	735.3	-	-	-1767.2	0.9	0.81	195.8	52.6	0.000	Significant
Ent_*SO*_	737.5	-	-	-1767.9	0.9	0.81	195.7	52.7	0.000	Significant
Ent_*R*_	739.9	-	-	-1767	0.9	0.82	194.2	53.6	0.000	Significant
**Quadratic**										
Ent_*ABC*_	-2467.1	499.9	-	3225.7	0.98	0.95	103.6	109.8	0.000	Significant
Ent_*F*_	-2493	511.9	-	3216.8	0.98	0.95	102.6	112	0.000	Significant
Ent_*GA*_	-2471.7	500.4	-	3234.4	0.98	0.95	103.3	110.5	0.000	Significant
Ent_*H*_	-2468.8	502.4	-	3214.5	0.98	0.95	102.8	111.6	0.000	Significant
Ent_*ISI*_	-2401.5	493.5	-	3105.2	0.97	0.95	107.4	101.7	0.000	Significant
${\mathrm{Ent}}_{M_{1}}$	-2472.4	502.8	-	3221	0.98	0.95	103.4	110.2	0.000	Significant
${\mathrm{Ent}}_{M_{2}}$	-2493.8	516.3	-	3194.3	0.97	0.94	111.9	93.3	0.000	Significant
Ent_*S*_	-2464.9	499.8	-	3220.7	0.98	0.95	103	111.2	0.000	Significant
Ent_*SO*_	-2464.8	501.4	-	3210.4	0.98	0.95	103.8	109.4	0.000	Significant
Ent_*R*_	-2428	497.4	-	3143.6	0.98	0.95	101.4	114.9	0.000	Significant
**Cubic**										
Ent_*ABC*_	2.8	-302.5	85.1	757.8	0.98	0.96	100	118.4	0.000	Significant
Ent_*F*_	4.4	-309.8	88.3	756.7	0.98	0.96	98.2	122.8	0.000	Significant
Ent_*GA*_	2.7	-302.2	85	757.4	0.98	0.96	99.7	119	0.000	Significant
Ent_*H*_	2.6	-302.7	85.6	751.2	0.98	0.96	99.3	120.1	0.000	Significant
Ent_*ISI*_	3.3	-296.9	84.7	732.8	0.98	0.95	103.6	109.8	0.000	Significant
${\mathrm{Ent}}_{M_{1}}$	2.8	-304.1	85.8	756.4	0.98	0.96	99.7	119	0.000	Significant
${\mathrm{Ent}}_{M_{2}}$	4.6	-320.3	91.4	778.8	0.97	0.95	107.2	102.2	0.000	Significant
Ent_*S*_	2.6	-301.5	84.9	753.7	0.98	0.96	99.4	119.7	0.000	Significant
Ent_*SO*_	2.6	-301.6	85.3	748.6	0.98	0.96	100.4	117.4	0.000	Significant
Ent_*R*_	2.1	-295.3	84.4	724.6	0.98	0.96	98.3	122.4	0.000	Significant

**Table 7:** Statistical parameters for the MV.

Entropy Index	$b_{1}$	$b_{2}$	$b_{3}$	a	r	$r^{2}$	SE	F	p-value	Indicator
**Exponential**										
Ent_*ABC*_	0.8	-	-	15.9	0.92	0.84	0.2	63.9	0.000	Significant
Ent_*F*_	0.8	-	-	18.6	0.89	0.8	0.2	47.5	0.000	Significant
Ent_*GA*_	0.8	-	-	15.8	0.92	0.84	0.2	63.9	0.000	Significant
Ent_*H*_	0.8	-	-	16	0.92	0.84	0.2	62.7	0.000	Significant
Ent_*ISI*_	0.8	-	-	16.2	0.92	0.84	0.2	64.7	0.000	Significant
${\mathrm{Ent}}_{M_{1}}$	0.8	-	-	16	0.92	0.84	0.2	62.9	0.000	Significant
${\mathrm{Ent}}_{M_{2}}$	0.8	-	-	16.8	0.92	0.84	0.2	62.1	0.000	Significant
Ent_*S*_	0.8	-	-	15.9	0.92	0.84	0.2	63.6	0.000	Significant
Ent_*SO*_	0.8	-	-	15.9	0.92	0.84	0.2	62.9	0.000	Significant
Ent_*R*_	0.9	-	-	16	0.92	0.84	0.2	63.3	0.000	Significant
**Linear**										
Ent_*ABC*_	222.1	-	-	-444	0.89	0.8	62.4	47.2	0.000	Significant
Ent_*F*_	213.8	-	-	-405.8	0.87	0.76	67.3	38.9	0.000	Significant
Ent_*GA*_	222.7	-	-	-446.3	0.89	0.8	62.3	47.5	0.000	Significant
Ent_*H*_	222.7	-	-	-443.3	0.89	0.8	62.6	46.9	0.000	Significant
Ent_*ISI*_	222.6	-	-	-439.2	0.9	0.8	61.8	48.4	0.000	Significant
${\mathrm{Ent}}_{M_{1}}$	222.4	-	-	-442.5	0.89	0.8	62.7	46.8	0.000	Significant
${\mathrm{Ent}}_{M_{2}}$	221.8	-	-	-427.4	0.89	0.79	63.5	45.2	0.000	Significant
Ent_*S*_	222.3	-	-	-444.5	0.89	0.8	62.4	47.2	0.000	Significant
Ent_*SO*_	222.9	-	-	-444.4	0.89	0.8	62.5	47.1	0.000	Significant
Ent_*R*_	223.5	-	-	-443.7	0.89	0.8	62.2	47.6	0.000	Significant
**Quadratic**										
Ent_*ABC*_	-582.8	125.7	-	810.5	0.94	0.89	47.7	45.2	0.000	Significant
Ent_*F*_	-688.9	143.8	-	970.8	0.94	0.88	49.4	41.8	0.000	Significant
Ent_*GA*_	-583.9	125.8	-	812.5	0.94	0.89	47.6	45.4	0.000	Significant
Ent_*H*_	-588.6	127.1	-	816.9	0.94	0.89	47.8	45.1	0.000	Significant
Ent_*ISI*_	-563	123.6	-	775.5	0.94	0.89	47.7	45.3	0.000	Significant
${\mathrm{Ent}}_{M_{1}}$	-588.8	127.1	-	817.4	0.94	0.89	47.9	44.9	0.000	Significant
${\mathrm{Ent}}_{M_{2}}$	-597.9	131.2	-	817.7	0.94	0.89	48.6	43.4	0.000	Significant
Ent_*S*_	-583.7	125.9	-	811.7	0.94	0.89	47.7	45.3	0.000	Significant
Ent_*SO*_	-591.1	127.4	-	821	0.94	0.89	47.6	45.6	0.000	Significant
Ent_*R*_	-571	124.7	-	787.8	0.94	0.89	47.8	45.1	0.000	Significant
**Cubic**										
Ent_*ABC*_	15.6	-71.7	21.7	254.1	0.95	0.9	45.4	50.6	0.000	Significant
Ent_*F*_	17.1	-89.7	25.7	311.6	0.95	0.89	47	46.9	0.000	Significant
Ent_*GA*_	15.5	-71.6	21.7	253.9	0.95	0.9	45.3	50.7	0.000	Significant
Ent_*H*_	15.6	-72.7	22	256.1	0.95	0.9	45.5	50.4	0.000	Significant
Ent_*ISI*_	16	-69.5	21.5	245.4	0.95	0.9	45.4	50.6	0.000	Significant
${\mathrm{Ent}}_{M_{1}}$	15.8	-73	22.1	257	0.95	0.9	45.5	50.3	0.000	Significant
${\mathrm{Ent}}_{M_{2}}$	17.6	-77.6	23.6	264.9	0.95	0.9	46	49	0.000	Significant
Ent_*S*_	15.5	-71.7	21.7	254	0.95	0.9	45.4	50.6	0.000	Significant
Ent_*SO*_	15.6	-73	22.1	257	0.95	0.9	45.2	51	0.000	Significant
Ent_*R*_	15.1	-69.5	21.4	245	0.95	0.9	45.6	50.1	0.000	Significant

**Table 8:** Statistical parameters for the Polarizability

Entropy Index	$b_{1}$	$b_{2}$	$b_{3}$	a	r	$r^{2}$	SE	F	p-value	Indicator
**Exponential**										
Ent_*ABC*_	1.1		-	1	0.99	0.99	0.1	888	0.000	Significant
Ent_*F*_	1	-	-	1.1	0.99	0.97	0.1	439.4	0.000	Significant
Ent_*GA*_	1.1	-	-	1	0.99	0.99	0.1	871.8	0.000	Significant
Ent_*H*_	1.1	-	-	1	0.99	0.99	0.1	842.8	0.000	Significant
Ent_*ISI*_	1.1	-	-	1	0.99	0.99	0.1	868.8	0.000	Significant
${\mathrm{Ent}}_{M_{1}}$	1.1	-	-	1	0.99	0.99	0.1	858.6	0.000	Significant
${\mathrm{Ent}}_{M_{2}}$	1.1	-	-	1.1	0.99	0.99	0.1	911.9	0.000	Significant
Ent_*S*_	1.1	-	-	1	0.99	0.99	0.1	866.1	0.000	Significant
Ent_*SO*_	1.1	-	-	1	0.99	0.99	0.1	848.9	0.000	Significant
Ent_*R*_	1.1	-	-	1	0.99	0.99	0.1	867.3	0.000	Significant
**Linear**										
Ent_*ABC*_	36.6	-	-	-81.3	0.96	0.92	6	140.4	0.000	Significant
Ent_*F*_	35.7	-	-	-76.6	0.95	0.91	6.5	118.5	0.000	Significant
Ent_*GA*_	36.7	-	-	-81.7	0.96	0.92	5.9	142.2	0.000	Significant
Ent_*H*_	36.7	-	-	-81.3	0.96	0.92	6	140.3	0.000	Significant
Ent_*ISI*_	36.7	-	-	-80.4	0.96	0.92	5.9	146.2	0.000	Significant
${\mathrm{Ent}}_{M_{1}}$	36.7	-	-	-81.2	0.96	0.92	6	139.8	0.000	Significant
${\mathrm{Ent}}_{M_{2}}$	36.6	-	-	-78.8	0.96	0.92	6.1	132.1	0.000	Significant
Ent_*S*_	36.6	-	-	-81.4	0.96	0.92	6	140.8	0.000	Significant
Ent_*SO*_	36.8	-	-	-81.4	0.96	0.92	6	141	0.000	Significant
Ent_*R*_	36.8	-	-	-81.3	0.96	0.92	5.9	145	0.000	Significant
**Quadratic**										
Ent_*ABC*_	-70.3	16.7	-	85.3	1	0.99	2	682.3	0.000	Significant
Ent_*F*_	-77.9	18.1	-	96.7	0.99	0.99	2.4	470.6	0.000	Significant
Ent_*GA*_	-583.9	125.8	-	812.5	0.94	0.89	47.6	45.4	0.000	Significant
Ent_*H*_	-70.5	16.8	-	85.2	1	0.99	2	659.6	0.000	Significant
Ent_*ISI*_	-67.5	16.4	-	80.6	1	0.99	2	660.5	0.000	Significant
${\mathrm{Ent}}_{M_{1}}$	-70.7	16.8	-	85.6	1	0.99	2	660.9	0.000	Significant
${\mathrm{Ent}}_{M_{2}}$	-73	17.5	-	87.8	1	0.99	2.1	587.6	0.000	Significant
Ent_*S*_	-70.2	16.7	-	85.1	1	0.99	2	673.4	0.000	Significant
Ent_*SO*_	-70.5	16.8	-	85.3	1	0.99	2	678.9	0.000	Significant
Ent_*R*_	-68.4	16.5	-	81.7	1	0.99	1.9	729.5	0.000	Significant
**Cubic**										
Ent_*ABC*_	2.3	-6.2	2.4	15.2	1	0.99	1.8	845.6	0.000	Significant
Ent_*F*_	3.7	-7.6	2.8	20	1	0.99	2.2	575.7	0.000	Significant
Ent_*GA*_	2.3	-6.2	2.4	15	1	0.99	1.8	838	0.000	Significant
Ent_*H*_	2.3	-6.2	2.5	15.1	1	0.99	1.8	806.9	0.000	Significant
Ent_*ISI*_	2.7	-5.9	2.4	14.1	1	0.99	1.8	820.7	0.000	Significant
${\mathrm{Ent}}_{M_{1}}$	2.5	-6.3	2.5	15.4	1	0.99	1.8	819.5	0.000	Significant
${\mathrm{Ent}}_{M_{2}}$	4	-7.1	2.7	17.4	1	0.99	1.8	791	0.000	Significant
Ent_*S*_	2.2	-6.2	2.4	15	1	0.99	1.8	825.4	0.000	Significant
Ent_*SO*_	2.3	-6.2	2.5	15.1	1	0.99	1.8	833.1	0.000	Significant
Ent_*R*_	1.7	-5.8	2.4	13.8	1	0.99	1.8	868.9	0.000	Significant

**Table 9:** Statistical parameters for the Molar Refractivity.

Entropy Index	$b_{1}$	$b_{2}$	$b_{3}$	a	r	$r^{2}$	SE	F	p-value	Indicator
**Exponential**										
Ent_*ABC*_	1	-	-	3.5	0.98	0.95	0.1	252.1	0.000	Significant
Ent_*F*_	1	-	-	4	0.96	0.93	0.2	151.5	0.000	Significant
Ent_*GA*_	1	-	-	3.4	0.98	0.95	0.1	251.5	0.000	Significant
Ent_*H*_	1	-	-	3.5	0.98	0.95	0.1	243.9	0.000	Significant
Ent_*ISI*_	1	-	-	3.5	0.98	0.96	0.1	260	0.000	Significant
${\mathrm{Ent}}_{M_{1}}$	1	-	-	3.5	0.98	0.95	0.1	245.9	0.000	Significant
${\mathrm{Ent}}_{M_{2}}$	1	-	-	3.7	0.98	0.95	0.1	247.6	0.000	Significant
Ent_*S*_	1	-	-	3.4	0.98	0.95	0.1	249	0.000	Significant
Ent_*SO*_	1	-	-	3.5	0.98	0.95	0.1	241.8	0.000	Significant
Ent_*R*_	1	-	-	3.5	0.98	0.95	0.1	252.6	0.000	Significant
**Linear**										
Ent_*ABC*_	89.2	-	-	-192	0.95	0.9	16.4	110.3	0.000	Significant
Ent_*F*_	86.7	-	-	-179.2	0.94	0.88	18.1	88.5	0.000	Significant
Ent_*GA*_	89.4	-	-	-192.9	0.95	0.9	16.3	111.5	0.000	Significant
Ent_*H*_	89.5	-	-	-191.9	0.95	0.9	16.5	109.7	0.000	Significant
Ent_*ISI*_	89.4	-	-	-190	0.95	0.91	16.1	115.7	0.000	Significant
${\mathrm{Ent}}_{M_{1}}$	89.4	-	-	-191.6	0.95	0.9	16.5	109.5	0.000	Significant
${\mathrm{Ent}}_{M_{2}}$	89.3	-	-	-185.9	0.95	0.9	16.8	104.7	0.000	Significant
Ent_*S*_	89.3	-	-	-192.2	0.95	0.9	16.4	110.4	0.000	Significant
Ent_*SO*_	89.5	-	-	-192.1	0.95	0.9	16.5	109.5	0.000	Significant
Ent_*R*_	89.8	-	-	-192.1	0.95	0.9	16.2	113.9	0.000	Significant
**Quadratic**										
Ent_*ABC*_	-180.7	42.1	-	228.7	0.99	0.98	8.5	224.8	0.000	Significant
Ent_*F*_	-209.6	47.2	-	272.6	0.99	0.97	9.4	182	0.000	Significant
Ent_*GA*_	-180.4	42.1	-	228.2	0.99	0.98	8.5	225.2	0.000	Significant
Ent_*H*_	-181.6	42.5	-	229.3	0.99	0.98	8.5	221.6	0.000	Significant
Ent_*ISI*_	-173.9	41.4	-	217.2	0.99	0.98	8.2	237.4	0.000	Significant
${\mathrm{Ent}}_{M_{1}}$	-182.1	42.5	-	230.2	0.99	0.98	8.5	222.1	0.000	Significant
${\mathrm{Ent}}_{M_{2}}$	-188.6	44.5	-	236.3	0.99	0.98	8.6	219	0.000	Significant
Ent_*S*_	-180.5	42.1	-	228.3	0.99	0.98	8.5	223.3	0.000	Significant
Ent_*SO*_	-181.6	42.5	-	229.4	0.99	0.98	8.6	218	0.000	Significant
Ent_*R*_	-176.4	41.8	-	220.6	0.99	0.98	8.2	238.5	0.000	Significant
**Cubic**										
Ent_*ABC*_	4.4	-17.2	6.3	49.9	0.99	0.98	8	253.3	0.000	Significant
Ent_*F*_	5.3	-22.3	7.5	67	0.99	0.97	8.9	203.8	0.000	Significant
Ent_*GA*_	4.3	-17.1	6.3	49.4	0.99	0.98	8	252.9	0.000	Significant
Ent_*H*_	4.3	-17.3	6.4	50	0.99	0.98	8.1	248.8	0.000	Significant
Ent_*ISI*_	4.6	-16.4	6.3	47.3	0.99	0.98	7.7	269.3	0.000	Significant
${\mathrm{Ent}}_{M_{1}}$	4.5	-17.5	6.4	50.6	0.99	0.98	8	250.6	0.000	Significant
${\mathrm{Ent}}_{M_{2}}$	6.1	-19.5	7.1	55.8	0.99	0.98	7.9	257.3	0.000	Significant
Ent_*S*_	4.3	-17.1	6.3	49.6	0.99	0.98	8	250.6	0.000	Significant
Ent_*SO*_	4.3	-17.3	6.4	50	0.99	0.98	8.1	244.1	0.000	Significant
Ent_*R*_	3.7	-16.3	6.2	46.6	0.99	0.98	7.8	265.3	0.000	Significant

**Table 10:** Statistical parameters for the logarithmic model.

Entropy Index	$b_{1}$	$b_{2}$	$b_{3}$	a	r	$r^{2}$	SE	F	p-value	Indicator
**Molecular Weight**										
Ent_*ABC*_	876.5	-	-	-607.9	0.86	0.74	92.1	34.8	0.000	Significant
Ent_*F*_	819.7	-	-	-527.3	0.84	0.7	99.8	27.9	0.0002	Significant
Ent_*GA*_	880.3	-	-	-613	0.86	0.75	91.9	35.1	0.0001	Significant
Ent_*H*_	875.9	-	-	-604.1	0.86	0.74	92.3	34.6	0.0001	Significant
Ent_*ISI*_	872.7	-	-	-595.3	0.87	0.75	91.1	35.8	0.0001	Significant
${\mathrm{Ent}}_{M_{1}}$	874.8	-	-	-602.8	0.86	0.74	92.4	34.6	0.0001	Significant
${\mathrm{Ent}}_{M_{2}}$	852.6	-	-	-560.7	0.86	0.73	93.7	33.2	0.0001	Significant
Ent_*S*_	877.4	-	-	-608.8	0.86	0.74	92.1	34.8	0.0001	Significant
Ent_*SO*_	876.6	-	-	-605.5	0.86	0.74	92.4	34.5	0.0001	Significant
Ent_*R*_	876.4	-	-	-602	0.74	0.72	92	34.9	0.0001	Significant
**Complexity**										
Ent_*ABC*_	2183.7	-	-	-1922.1	0.86	0.74	231.1	34.3	0.0001	Significant
Ent_*F*_	2107.6	-	-	-1796.6	0.86	0.74	230.6	34.5	0.0001	Significant
Ent_*GA*_	2193.9	-	-	-1935.3	0.86	0.74	230.3	34.7	0.0001	Significant
Ent_*H*_	2186.2	-	-	-1917	0.86	0.74	230.4	34.6	0.0001	Significant
Ent_*ISI*_	2168.2	-	-	-1883.5	0.86	0.74	230.5	34.6	0.0001	Significant
${\mathrm{Ent}}_{M_{1}}$	2182.6	-	-	-1913	0.86	0.74	230.7	34.5	0.0001	Significant
${\mathrm{Ent}}_{M_{2}}$	2123.5	-	-	-1803.5	0.86	0.73	235.2	32.7	0.0001	Significant
Ent_*S*_	2187.3	-	-	-1925.6	0.86	0.74	230.6	34.5	0.0001	Significant
Ent_*SO*_	2189	-	-	-1921.6	0.86	0.74	230.4	34.6	0.0001	Significant
Ent_*R*_	2187.7	-	-	-1912.2	0.86	0.74	229.6	35	0.0001	Significant
**Molar Volume**										
Ent_*ABC*_	666.7	-	-	-499	0.86	0.74	70.7	34.2	0.0001	Significant
Ent_*F*_	627.2	-	-	-441.9	0.84	0.7	75.4	28.6	0.0002	Significant
Ent_*GA*_	669.6	-	-	-502.8	0.86	0.74	70.5	34.5	0.0001	Significant
Ent_*H*_	666.2	-	-	-495.9	0.86	0.74	70.9	34	0.0001	Significant
Ent_*ISI*_	663.3	-	-	-488.7	0.86	0.74	70.1	35	0.0001	Significant
${\mathrm{Ent}}_{M_{1}}$	665.4	-	-	-495	0.86	0.74	70.9	34	0.0001	Significant
${\mathrm{Ent}}_{M_{2}}$	649.1	-	-	-463.6	0.86	0.73	71.7	32.9	0.0001	Significant
Ent_*S*_	667.3	-	-	-499.5	0.86	0.74	70.7	34.2	0.0001	Significant
Ent_*SO*_	667.3	-	-	-497.7	0.86	0.74	70.8	34.1	0.0001	Significant
Ent_*R*_	666.9	-	-	-494.8	0.86	0.74	70.5	34.4	0.0001	Significant
**Polarizability**										
Ent_*ABC*_	110.4	-	-	-91	0.93	0.86	7.9	75.8	0.000	Significant
Ent_*F*_	105.7	-	-	-83.6	0.92	0.85	8.2	67.9	0.000	Significant
Ent_*GA*_	110.9	-	-	-91.6	0.93	0.86	7.8	76.7	0.000	Significant
Ent_*H*_	110.4	-	-	-90.6	0.93	0.86	7.9	75.9	0.000	Significant
Ent_*ISI*_	109.7	-	-	-89.2	0.93	0.87	7.8	78	0.000	Significant
${\mathrm{Ent}}_{M_{1}}$	110.3	-	-	-90.5	0.93	0.86	7.9	75.7	0.000	Significant
${\mathrm{Ent}}_{M_{2}}$	107.7	-	-	-85.4	0.93	0.86	8	72.5	0.000	Significant
Ent_*S*_	110.5	-	-	-91.1	0.93	0.86	7.9	76	0.000	Significant
Ent_*SO*_	110.6	-	-	-90.9	0.93	0.86	7.8	76.2	0.000	Significant
Ent_*R*_	110.5	-	-	-90.4	0.93	0.87	7.8	77.2	0.000	Significant

**Table 11:** Correlation coefficient for logarithmic, exponential, linear, quadratic and cubic regression models.

Logarithmic								Exponential						
EI	MW	TSA	C	D	MV	P	MR	MW	TSA	C	D	MV	P	MR
Ent_*ABC*_	0.86	0.53	0.86	0.41	0.86	0.93	0.92	0.9	0.58	0.91	0.46	0.92	0.99	0.98
Ent_*F*_	0.84	0.53	0.86	0.42	0.84	0.92	0.91	0.87	0.58	0.92	0.46	0.89	0.99	0.96
Ent_*GA*_	0.86	0.53	0.86	0.41	0.86	0.93	0.92	0.9	0.57	0.91	0.46	0.92	0.99	0.98
Ent_*H*_	0.86	0.53	0.86	0.4	0.86	0.93	0.92	0.9	0.58	0.91	0.45	0.92	0.99	0.98
Ent_*ISI*_	0.87	0.53	0.86	0.41	0.86	0.93	0.92	0.9	0.58	0.91	0.46	0.92	0.99	0.98
${\mathrm{Ent}}_{M_{1}}$	0.86	0.53	0.86	0.4	0.86	0.93	0.92	0.9	0.58	0.91	0.46	0.92	0.99	0.98
${\mathrm{Ent}}_{M_{2}}$	0.86	0.52	0.86	0.41	0.86	0.93	0.92	0.9	0.57	0.91	0.46	0.92	0.99	0.98
Ent_*S*_	0.86	0.53	0.86	0.41	0.86	0.93	0.92	0.9	0.58	0.91	0.46	0.92	0.99	0.98
Ent_*SO*_	0.86	0.53	0.86	0.41	0.86	0.93	0.92	0.9	0.57	0.91	0.46	0.92	0.99	0.98
Ent_*R*_	0.74	0.53	0.86	0.41	0.86	0.93	0.92	0.9	0.58	0.91	0.46	0.92	0.99	0.98
**Linear**								**Quadratic**						
**EI**	**MW**	**TSA**	**C**	**D**	**MV**	**P**	**MR**	**MW**	**TSA**	**C**	**D**	**MV**	**P**	**MR**
Ent_*ABC*_	0.89	0.54	0.9	0.44	0.89	0.96	0.95	0.93	0.54	0.98	0.52	0.94	1	0.99
Ent_*F*_	0.87	0.54	0.9	0.44	0.87	0.95	0.94	0.93	0.54	0.98	0.51	0.94	0.99	0.99
Ent_*GA*_	0.89	0.54	0.9	0.44	0.89	0.96	0.95	0.93	0.54	0.98	0.52	0.94	0.94	0.99
Ent_*H*_	0.89	0.54	0.9	0.44	0.89	0.96	0.95	0.93	0.54	0.98	0.52	0.94	1	0.99
Ent_*ISI*_	0.89	0.54	0.9	0.44	0.9	0.96	0.95	0.93	0.54	0.97	0.52	0.94	1	0.99
${\mathrm{Ent}}_{M_{1}}$	0.89	0.54	0.9	0.44	0.89	0.96	0.95	0.93	0.54	0.98	0.52	0.94	1	0.99
${\mathrm{Ent}}_{M_{2}}$	0.89	0.53	0.9	0.44	0.89	0.96	0.95	0.93	0.53	0.97	0.52	0.94	1	0.99
Ent_*S*_	0.89	0.54	0.9	0.44	0.89	0.96	0.95	0.93	0.54	0.98	0.52	0.94	1	0.99
Ent_*SO*_	0.89	0.53	0.9	0.44	0.89	0.96	0.95	0.93	0.54	0.98	0.52	0.94	1	0.99
Ent_*R*_	0.79	0.54	0.9	0.44	0.89	0.96	0.95	0.87	0.55	0.98	0.52	0.94	1	0.99
**Cubic**														
**EI**	**MW**	**TSA**	**C**	**D**	**MV**	**P**	**MR**							
Ent_*ABC*_	0.94	0.54	0.98	0.51	0.95	1	0.99							
Ent_*F*_	0.93	0.55	0.98	0.5	0.95	1	0.99							
Ent_*GA*_	0.94	0.54	0.98	0.51	0.95	1	0.99							
Ent_*H*_	0.94	0.55	0.98	0.51	0.95	1	0.99							
Ent_*ISI*_	0.94	0.54	0.98	0.51	0.95	1	0.99							
${\mathrm{Ent}}_{M_{1}}$	0.94	0.54	0.98	0.51	0.95	1	0.99							
${\mathrm{Ent}}_{M_{2}}$	0.93	0.54	0.97	0.52	0.95	1	0.99							
Ent_*S*_	0.94	0.55	0.98	0.51	0.95	1	0.99							
Ent_*SO*_	0.94	0.54	0.98	0.51	0.95	1	0.99							
Ent_*R*_	0.88	0.55	0.98	0.51	0.95	1	0.99							

**Table 12:** Coefficient of determination for logarithmic, exponential, linear, quadratic and cubic regression models.

Logarithmic								Exponential						
EI	MW	TSA	C	D	MV	P	MR	MW	TSA	C	D	MV	P	MR
Ent_*ABC*_	0.744	0.278	0.741	0.165	0.74	0.863	0.844	0.808	0.331	0.824	0.208	0.842	0.987	0.955
Ent_*F*_	0.699	0.279	0.742	0.174	0.704	0.85	0.821	0.759	0.332	0.846	0.214	0.798	0.973	0.927
Ent_*GA*_	0.745	0.278	0.743	0.165	0.742	0.865	0.846	0.808	0.33	0.824	0.208	0.842	0.986	0.954
Ent_*H*_	0.743	0.279	0.743	0.164	0.739	0.863	0.844	0.806	0.331	0.826	0.207	0.839	0.986	0.953
Ent_*ISI*_	0.749	0.28	0.742	0.166	0.745	0.867	0.849	0.811	0.331	0.82	0.209	0.843	0.986	0.956
${\mathrm{Ent}}_{M_{1}}$	0.742	0.279	0.742	0.164	0.739	0.863	0.844	0.806	0.331	0.826	0.207	0.84	0.986	0.953
${\mathrm{Ent}}_{M_{2}}$	0.735	0.274	0.732	0.168	0.733	0.858	0.838	0.803	0.326	0.821	0.212	0.838	0.987	0.954
Ent_*S*_	0.744	0.279	0.742	0.164	0.74	0.864	0.844	0.808	0.331	0.825	0.207	0.841	0.986	0.954
Ent_*SO*_	0.742	0.277	0.743	0.166	0.74	0.864	0.844	0.805	0.329	0.826	0.209	0.84	0.986	0.953
Ent_*R*_	0.744	0.283	0.744	0.165	0.741	0.865	0.847	0.807	0.336	0.824	0.209	0.841	0.986	0.955
**Linear**								**Quadratic**						
**EI**	**MW**	**TSA**	**C**	**D**	**MV**	**P**	**MR**	**MW**	**TSA**	**C**	**D**	**MV**	**P**	**MR**
Ent_*ABC*_	0.794	0.287	0.813	0.192	0.797	0.921	0.902	0.867	0.293	0.952	0.269	0.892	0.992	0.976
Ent_*F*_	0.754	0.288	0.812	0.198	0.764	0.908	0.881	0.858	0.295	0.953	0.258	0.884	0.988	0.971
Ent_*GA*_	0.795	0.287	0.815	0.192	0.798	0.922	0.903	0.868	0.294	0.953	0.268	0.892	0.992	0.976
Ent_*H*_	0.793	0.288	0.815	0.19	0.796	0.921	0.901	0.867	0.295	0.953	0.266	0.891	0.992	0.976
Ent_*ISI*_	0.799	0.287	0.814	0.193	0.801	0.924	0.906	0.870	0.292	0.949	0.268	0.892	0.992	0.977
${\mathrm{Ent}}_{M_{1}}$	0.793	0.287	0.814	0.191	0.796	0.921	0.901	0.867	0.294	0.952	0.267	0.891	0.992	0.976
${\mathrm{Ent}}_{M_{2}}$	0.786	0.281	0.804	0.195	0.797	0.917	0.897	0.862	0.285	0.949	0.275	0.888	0.991	0.976
Ent_*S*_	0.794	0.287	0.814	0.191	0.797	0.921	0.902	0.868	0.294	0.953	0.268	0.892	0.992	0.976
Ent_*SO*_	0.792	0.285	0.814	0.193	0.797	0.922	0.901	0.866	0.291	0.952	0.271	0.892	0.992	0.975
Ent_*R*_	0.794	0.292	0.817	0.192	0.799	0.924	0.905	0.866	0.299	0.954	0.267	0.891	0.993	0.977
**Cubic**														
**EI**	**MW**	**TSA**	**C**	**D**	**MV**	**P**	**MR**							
Ent_*ABC*_	0.877	0.297	0.956	0.263	0.902	0.994	0.979							
Ent_*F*_	0.869	0.298	0.957	0.254	0.895	0.991	0.974							
Ent_*GA*_	0.878	0.297	0.956	0.262	0.902	0.993	0.979							
Ent_*H*_	0.878	0.298	0.956	0.260	0.902	0.993	0.978							
Ent_*ISI*_	0.880	0.295	0.952	0.262	0.902	0.993	0.980							
${\mathrm{Ent}}_{M_{1}}$	0.877	0.297	0.956	0.261	0.901	0.993	0.979							
${\mathrm{Ent}}_{M_{2}}$	0.874	0.287	0.949	0.269	0.899	0.993	0.979							
Ent_*S*_	0.878	0.298	0.956	0.262	0.902	0.993	0.979							
Ent_*SO*_	0.877	0.294	0.955	0.265	0.903	0.993	0.978							
Ent_*R*_	0.876	0.302	0.957	0.261	0.901	0.994	0.980							

### Strength and direction of relationship

- Molecular weight, complexity, molar volume, polarizability and molar refractivity has strong and positive linear relationship with entropy indices as corresponding values lies between 0.74 and 1.0 for all the regression models, but if we compare all the models, in case of cubic regression model all these properties has strong and positive relationship with all the entropy indices.
- Topological surface area and Density have weak relationship with all the entropy indices as *r*-values lies between 0.4 and 0.54 which are very small.

### Goodness of fit

- Molecular weight can be predicted using Ent_*ABC*_, Ent_*F*_, Ent_*GA*_, Ent_*H*_, Ent_*ISI*_, ${\mathrm{Ent}}_{M_{1}}$, ${\mathrm{Ent}}_{M_{2}}$, Ent_*S*_, Ent_*SO*_ or Ent_*R*_, as corresponding values of ${\text{r}}^{2}$ (coefficient of determination) lies between 0.70 and 0.88 for all the regression models, but if we compare all the models, the cubic regression model is the best predictive model for MW.
- Topological surface area and Density cannot be predicted by any model as values lies between 0.16 and 0.34 which are very small. So, TSA and density cannot be predicted using these entropy indices.
- Complexity can be predicted using Ent_*ABC*_, Ent_*F*_, Ent_*GA*_, Ent_*H*_, Ent_*ISI*_, ${\mathrm{Ent}}_{M_{1}}$, ${\mathrm{Ent}}_{M_{2}}$, Ent_*S*_, Ent_*SO*_ or Ent_*R*_, as corresponding values lies between 0.74 and 0.96 for all the regression models, but if we compare all the models, the cubic regression model is the best predictive model for complexity.
- Molar volume can be predicted using Ent_*ABC*_, Ent_*F*_, Ent_*GA*_, Ent_*H*_, Ent_*ISI*_, ${\mathrm{Ent}}_{M_{1}}$, ${\mathrm{Ent}}_{M_{2}}$, Ent_*S*_, Ent_*SO*_ or Ent_*R*_, as corresponding values lies between 0.70 and 0.90 for all the regression models, but if we compare all the models, the cubic regression model is the best predictive model for MV.
- Polarizability can be predicted using Ent_*ABC*_, Ent_*F*_, Ent_*GA*_, Ent_*H*_, Ent_*ISI*_, ${\mathrm{Ent}}_{M_{1}}$, ${\mathrm{Ent}}_{M_{2}}$, Ent_*S*_, Ent_*SO*_ or Ent_*R*_, as corresponding values lies between 0.85 and 0.99 for all the regression models, but if we compare all the models, the cubic regression model is the best predictive model for polarizability as the corresponding value is 1 showing the best fitted model.
- Molar refractivity can be predicted using Ent_*ABC*_, Ent_*F*_, Ent_*GA*_, Ent_*H*_, Ent_*ISI*_, ${\mathrm{Ent}}_{M_{1}}$, ${\mathrm{Ent}}_{M_{2}}$, Ent_*S*_, Ent_*SO*_ or Ent_*R*_, as corresponding values lies between 0.82 and 0.98 for all the regression models, but if we compare all the models, the cubic regression model is the best predictive model for *MR*.

Across all the models, cubic regression models is the best predictive model, for molecular weight, complexity, molar volume, polarizability and molar refractivity. But, quadratic models can also considered predictive model particularly for polarizability and molar refractivity.

**Limitations of regression models**

- Linear regression assumes a straight-line relationship, making it unsuitable for nonlinear data. Small samples can lead to underfitting, while large datasets improve accuracy for linear trends.
- Quadratic regression captures parabolic relationships but may overfit small datasets or under fit complex patterns. It struggles with data exhibiting multiple inflection points.
- Cubic regression models complex curves with inflection points but requires large datasets to avoid overfitting. It performs poorly on data without a clear cubic trend.
- Logarithmic regression fits diminishing return patterns but fails to model exponential growth. Small samples can result in unreliable parameter estimates.
- Exponential regression models rapid growth or decay but is sensitive to outliers. It performs poorly on noisy data or trends that deviate from exponential behavior. A cubic regression model includes a third-degree term (${\text{x}}^{3}$), allowing it to capture more complex trends, including multiple inflection points where the direction of the curve changes and a quadratic model is considered viable when it appropriately describes the relationship between the dependent and independent variable(s) in a given dataset.

**Statistical validation**

Robustness is assessed using statistical metrics such as the coefficient of determination (${\text{r}}^{2}$), root mean squared error (RMSE), and mean absolute error (MAE). A well-fitted model is characterized by high ${\text{r}}^{2}$ values, low error metrics, and narrow confidence intervals, which indicate precise parameter estimates. A 95% confidence interval (CI) provides a range of values within which we are 95% confident that the true population parameter (such as the mean or proportion) lies. The lower bound and upper bound define the limits of this range, values for constant and slope lies within the interval. An ${\text{r}}^{2}$ value of 0.85 suggests that the model explains 85% of the variance in the target property, demonstrating strong predictive performance. Additionally, metrics like adjusted ${\text{R}}^{2}$ are used to account for model complexity and avoid overfitting, while confidence intervals provide insight into the reliability of the model’s predictions.

**Fig 3:**
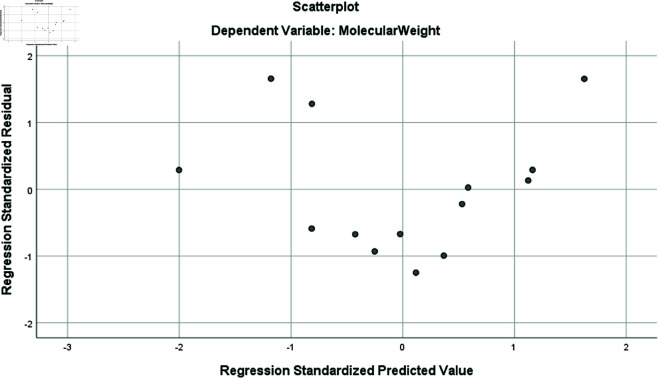
Residual plot for Molecular Weight for *Ent*_*ABC*_.

The adjusted ${\text{R}}^{2}$ is a modified version of ${\text{r}}^{2}$ that accounts for the number of predictors in the model. Its value ranges from 0 to 1, when its value close to 1: indicates a strong fit, with the model explaining most of the variance in the response variable while penalizing unnecessary predictors, and when it close to 0: suggests a poor fit, meaning the model explains little variance or includes too many irrelevant predictors. In our study, for molecular weight, complexity, molar volume, polarizability and molar refractivity values for adjusted ${\text{R}}^{2}$ are close to 1 but for topological surface area and density it is lose to zero indicating poor fit. Residual plots showed random scatter around zero, confirming that the QSPR model adequately captures the relationship between entropy indices and physio-chemical properties. The random scatter indicates that the model’s predictions are unbiased and that no systematic patterns or trends are present in the residuals. This supports the assumptions of linearity, homoscedasticity, and normality, ensuring the model’s reliability and validity as shown in Fig 3.

### Graphical representation

The graphical representation of comparison of correlation between entropy indices and physio-chemical properties for the colorectal cancer drugs is shown in Figs 4, 5, 6, 7, 8, 9, 10, 11, 12, and 13.

**Fig 4:**
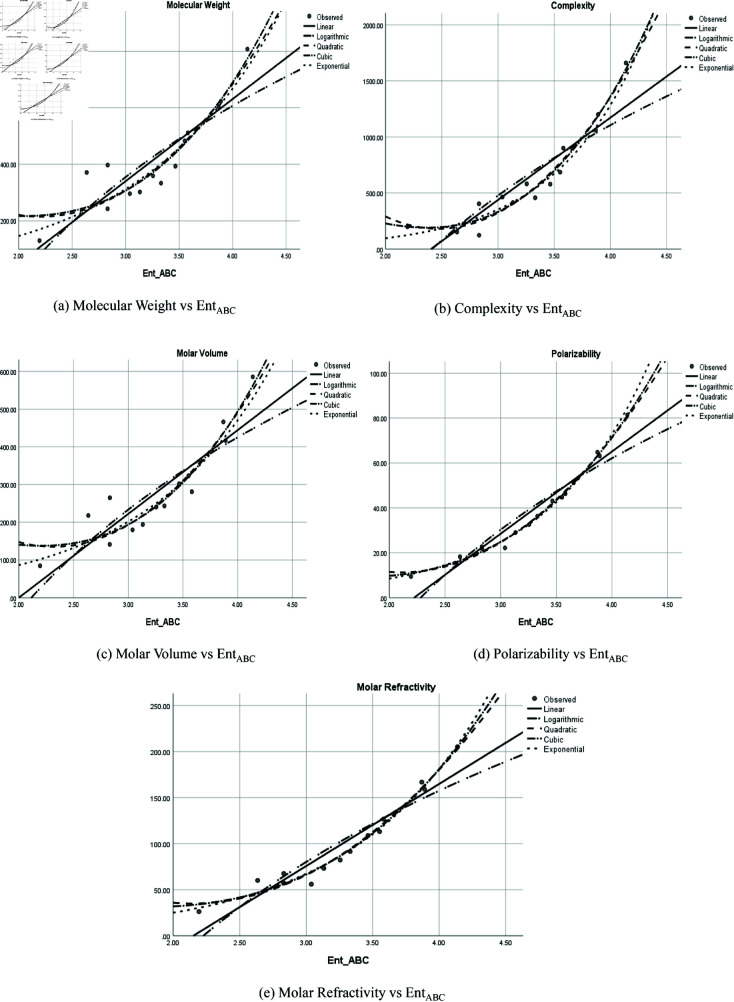
Comparison of correlation between entropy index *Ent*_*ABC*_ and physio-chemical properties for the colorectal cancer drugs.

**Fig 5:**
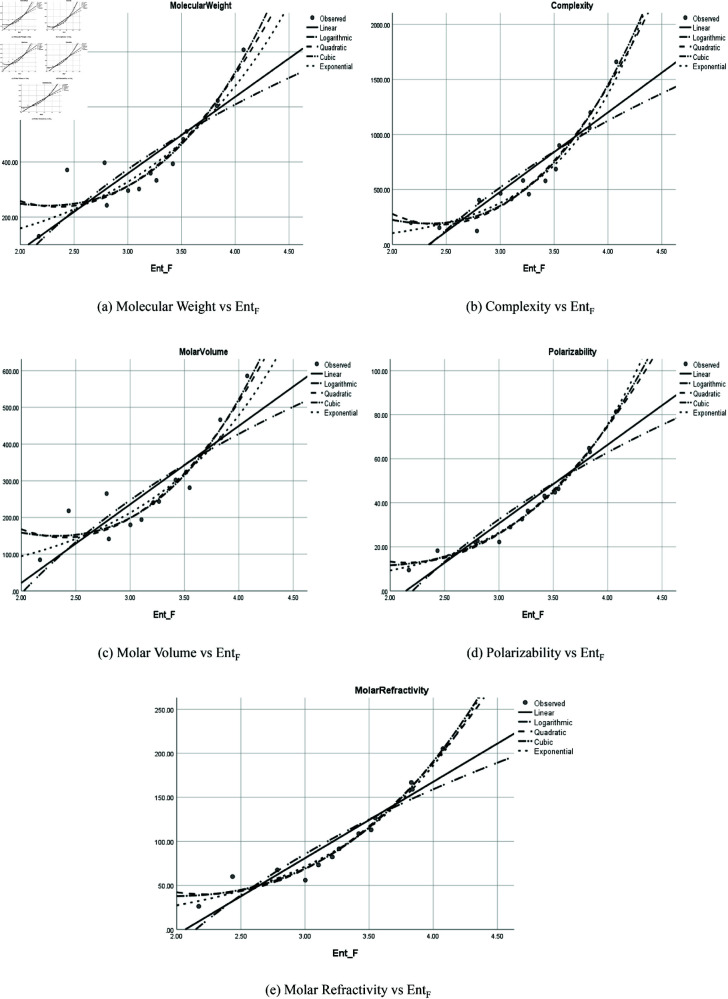
Comparison of correlation between entropy index *Ent*_*F*_ and physio-chemical properties for the colorectal cancer drugs.

**Fig 6:**
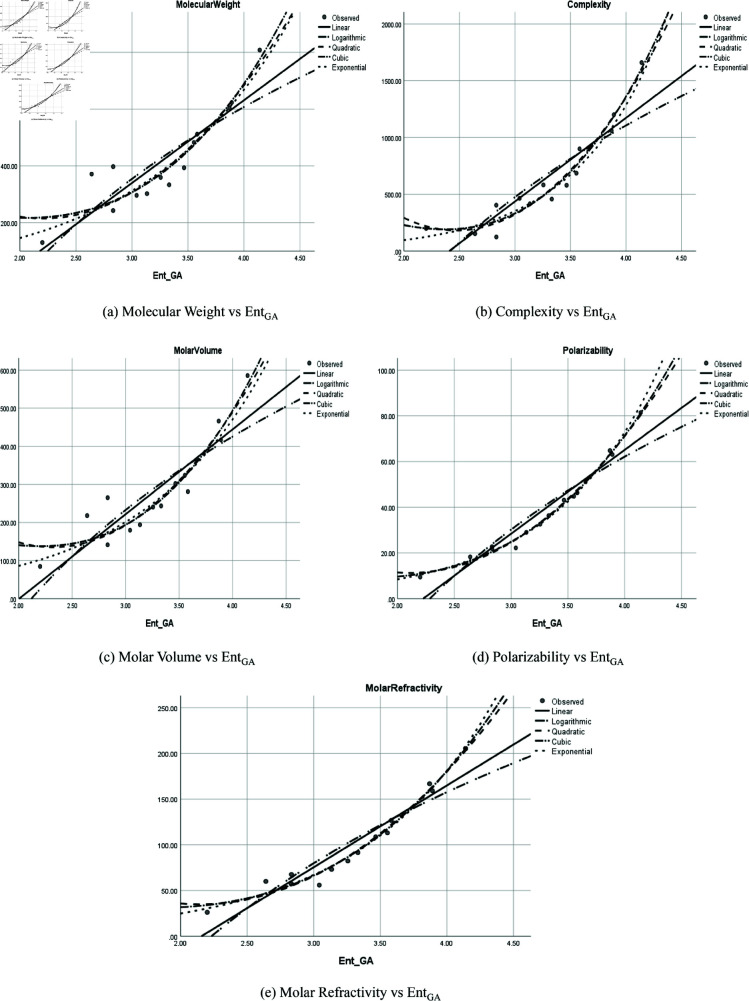
Comparison of correlation between entropy index *Ent*_*GA*_ and physio-chemical properties for the colorectal cancer drugs.

**Fig 7:**
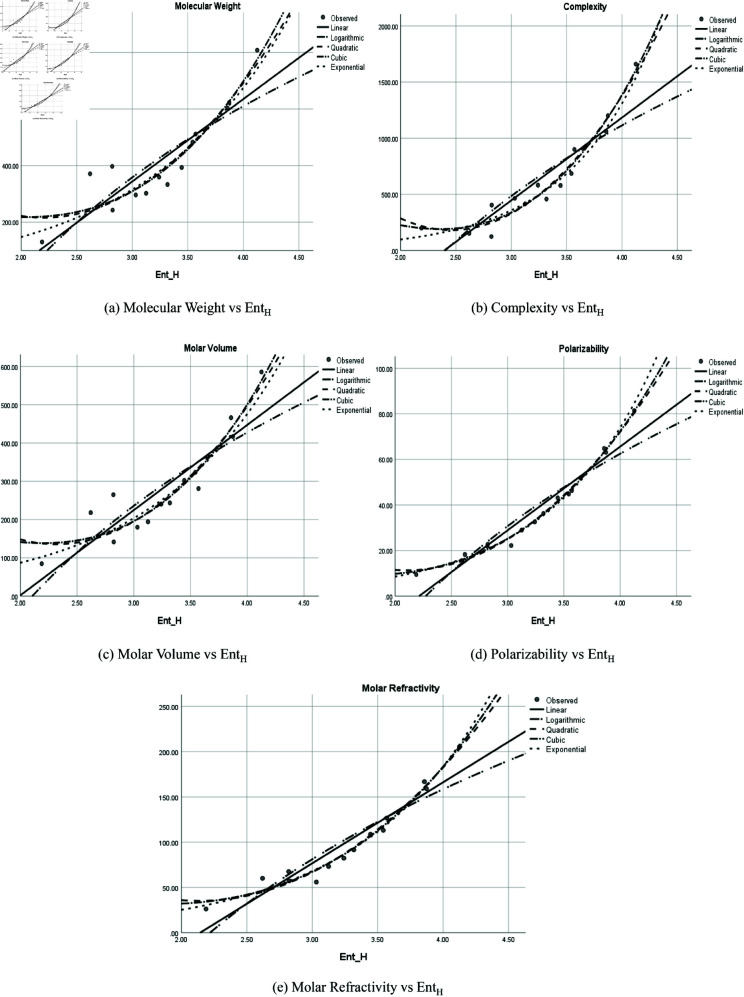
Comparison of correlation between entropy index *Ent*_*H*_ and physio-chemical properties for the colorectal cancer drugs.

**Fig 8:**
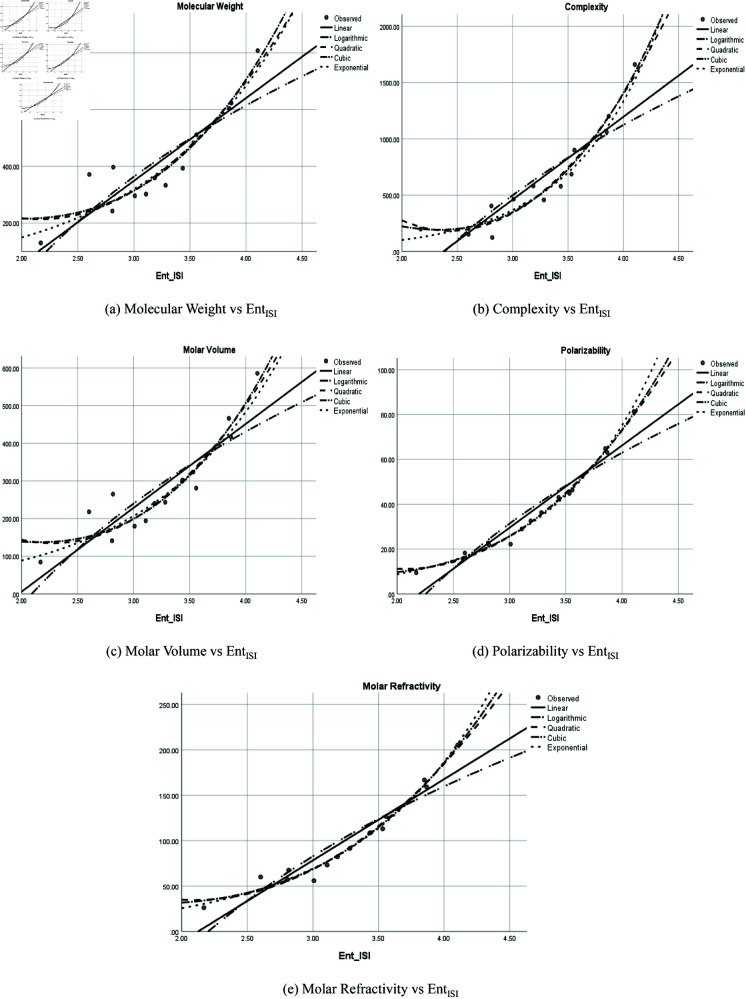
Comparison of correlation between entropy index *Ent*_*ISI*_ and physio-chemical properties for the colorectal cancer drugs.

**Fig 9:**
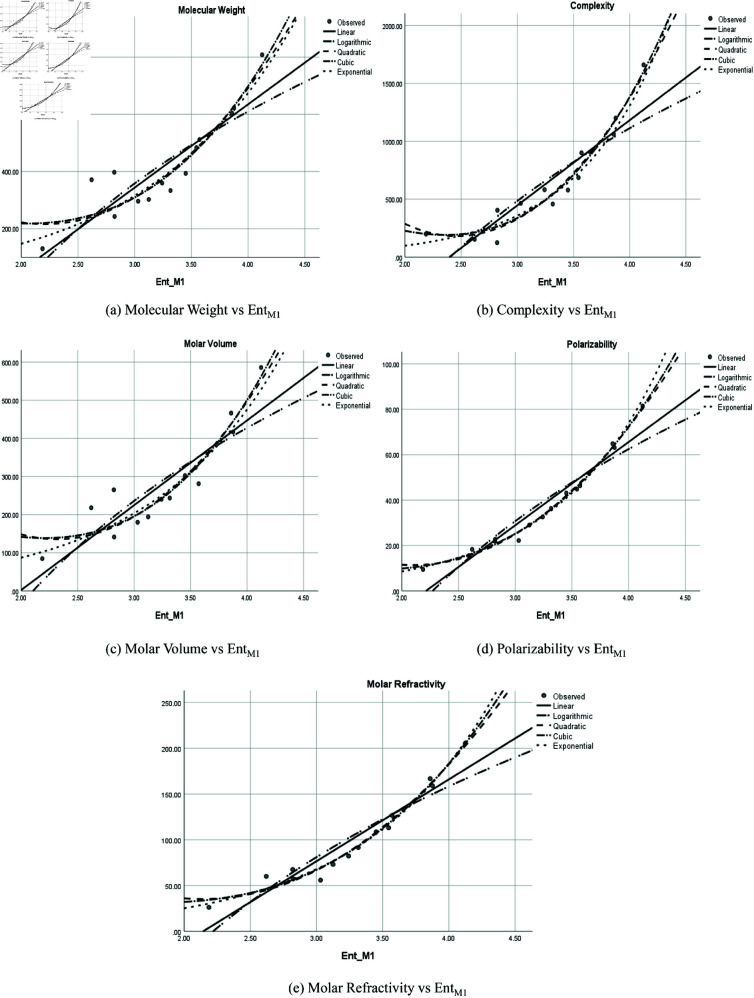
Comparison of correlation between entropy index $Ent_{M_{1}}$ and physio-chemical properties for the colorectal cancer drugs.

**Fig 10:**
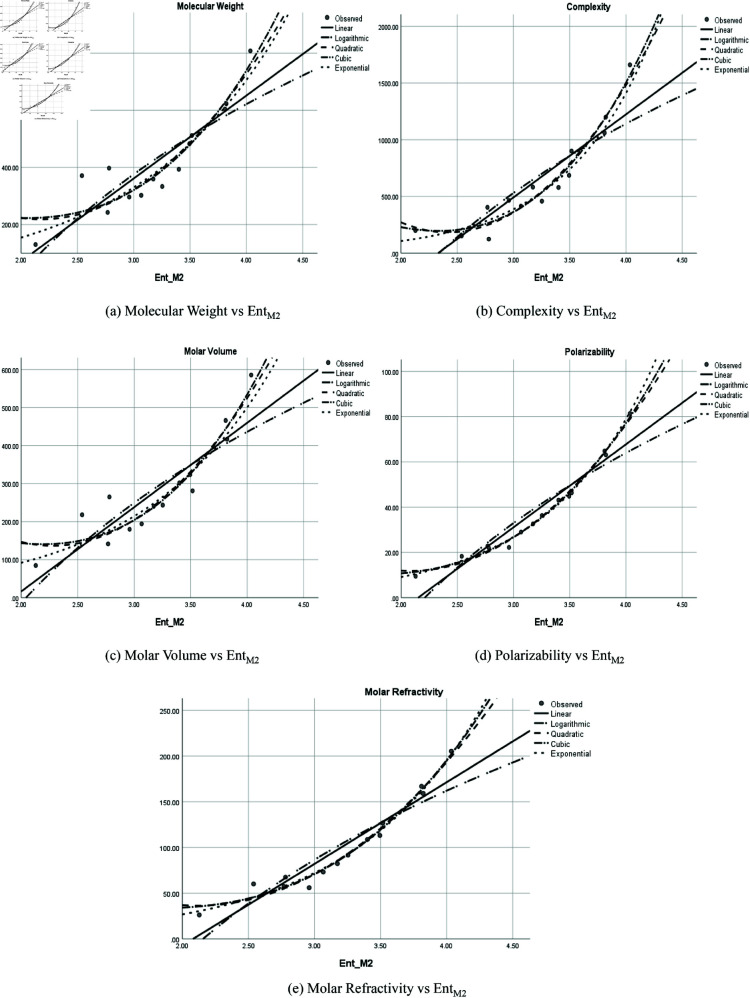
Comparison of correlation between entropy index $Ent_{M_{2}}$ and physio-chemical properties for the colorectal cancer drugs.

**Fig 11:**
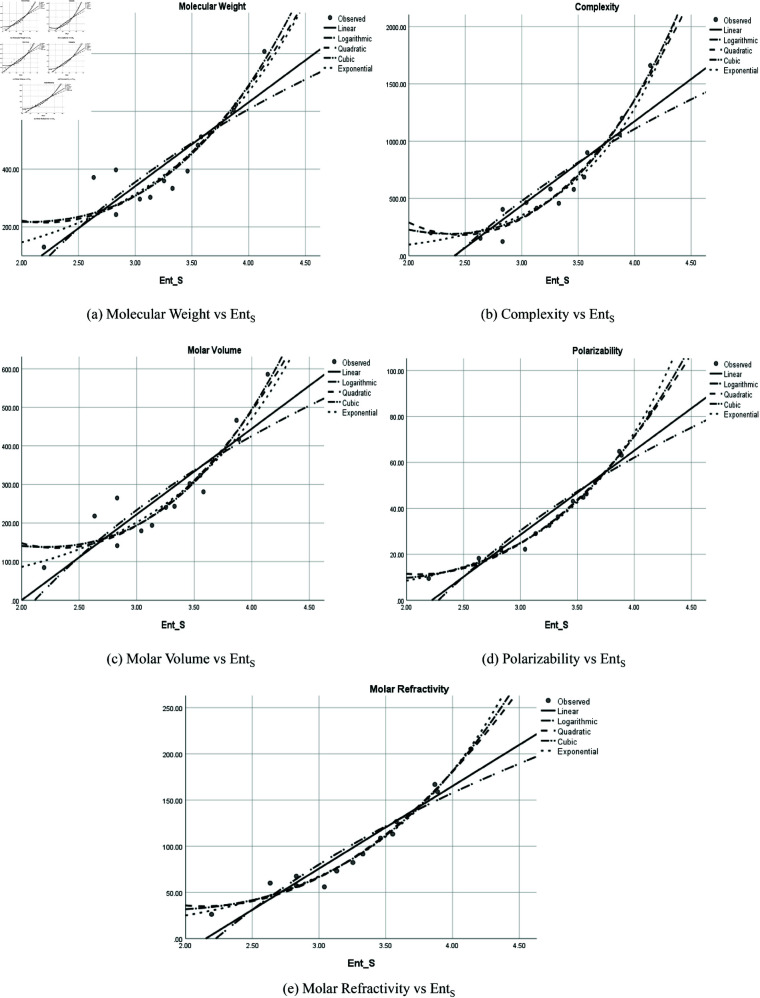
Comparison of correlation between entropy index *Ent*_*S*_ and physio-chemical properties for the colorectal cancer drugs.

**Fig 12:**
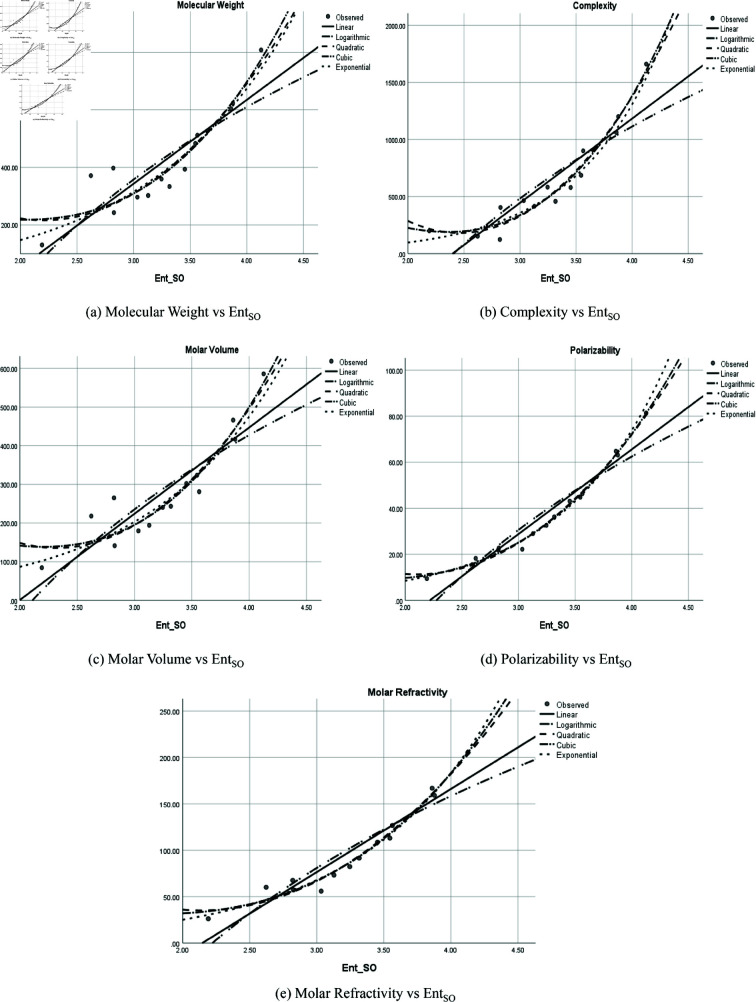
Comparison of correlation between entropy index *Ent*_*SO*_ and physio-chemical properties for the colorectal cancer drugs.

**Fig 13:**
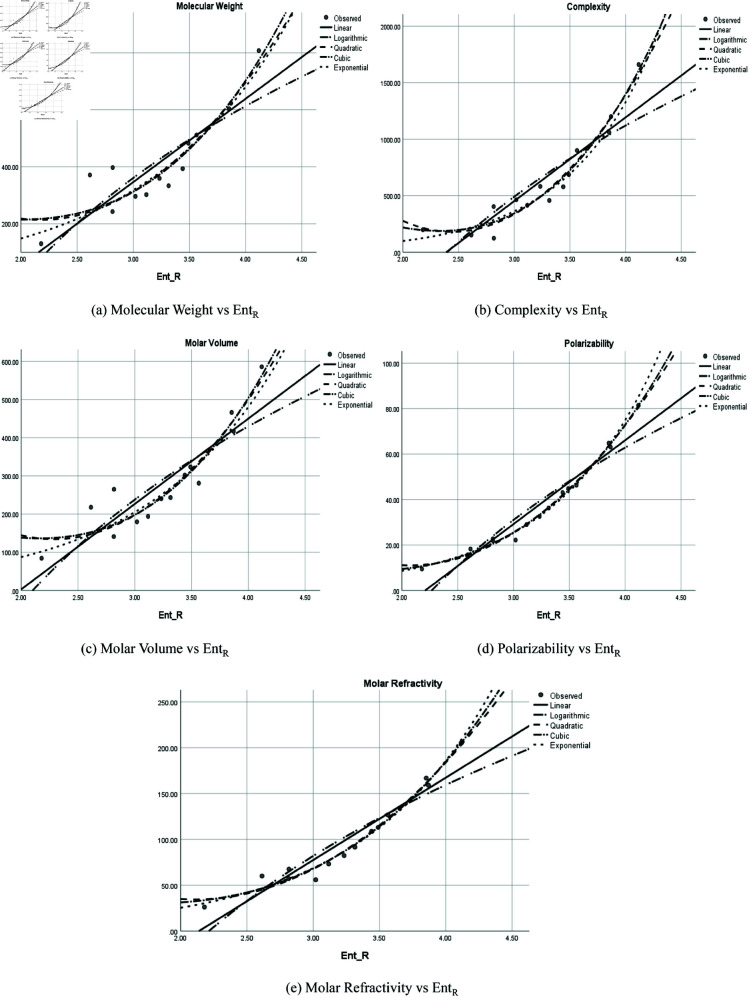
Comparison of correlation between entropy index *Ent*_*R*_ and physio-chemical properties for the colorectal cancer drugs.

## Validity of the regression model

**Fig 14:**
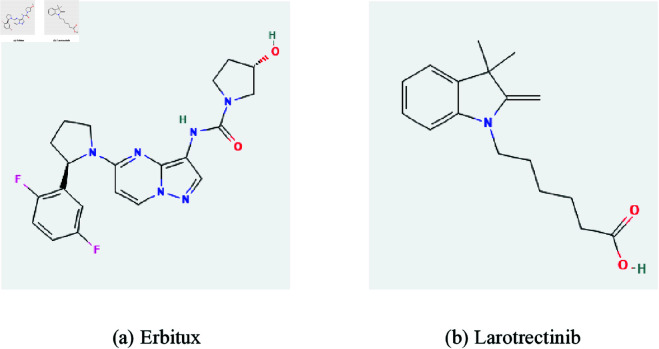
Molecular structure for Erbitux and Larotrectinib.

In order to check the validity of our results, we take the drugs, Erbitux and Larotrectinib shown in Fig 14. The computed values of the entropy indices for these drugs are given Table 13. The physicochemical properties of Erbitux and Larotrectinib are given in Table 14. The molecular structures and physio-chemical prperties are taken from PubChem and drugbank. We have computed the values of the physicochemical properties of Erbitux and Larotrectinib in order to compare with the experimental values. The comparison between experimental and calculated values is shown in Tables 15 and 16 respectively. The calculated values are obtained by using models which we have fitted in section 3 using SPSS and further we check the validity for properties under consideration. We have observed that, for molecular weight (MW), complexity (C), polarizability (P) and molar refractivity (MR) having coefficient of determination *r*^2^ = 0.880, *r*^2^ = 0.957, *r*^2^ = 0.994 and *r*^2^ = 0.980 respectively. It can be observed that the predicted value of these properties are closer to the experimental values. In case of complexity molar refractivity the best fitted models are cubic models corresponding to *R*(*G*).

**Table 13:** Calculated values of entropy indices of Erbitux and Larotrectinib.

Name of drugs	${\mathrm{Ent}}_{ABC}$	${\mathrm{Ent}}_{F}$	${\mathrm{Ent}}_{GA}$	${\mathrm{Ent}}_{H}$	${\mathrm{Ent}}_{ISI}$	${\mathrm{Ent}}_{M_{1}}$	${\mathrm{Ent}}_{M_{2}}$	${\mathrm{Ent}}_{S}$	${\mathrm{Ent}}_{SO}$	${\mathrm{Ent}}_{R}$
Erbitux	3.04	2.9432	3.042	3.028	3.01	3.025	2.939	3.04	3.025	3.024
Larotrectinib	3.55	3.524	3.55	3.54	3.538	3.545	3.505	3.55	3.547	3.54

**Table 14:** Physio-chemical properties of Erbitux and Larotrectinib.

Drugs name	Molecular Weight	Complexity	TSA	Polarizability	MR
Erbitux	273.37	375	40.5	27.5	69.5
Larotrectinib	428.4	659	86	41.61	122.96

**Table 15:** Comparison between experimental and calculated values of Erbitux.

Properties	Regression models	Experimental	Calculated
		values	values
MW	MW= 323.0 +18.4[Ent_*ISI*_*]*- 75.9[${\mathrm{Ent}}_{ISI}{]}^{2}$+24.7[${\mathrm{Ent}}_{ISI}{]}^{3}$	273.37	364.31
C	C=756.7+4.4(=[Ent_*F*_*]*-309.8[${\mathrm{Ent}}_{F}{]}^{2}$+88.3[${\mathrm{Ent}}_{F}{]}^{3}$	375	337.26
C	C = 724.6+2.1[Ent_*R*_*]*-295.3[${\mathrm{Ent}}_{R}{]}^{2}$+84.4[${\mathrm{Ent}}_{R}{]}^{3}$	375	364.48
P	P = 13.8+1.7[Ent_*R*_*]*-5.8[${\mathrm{Ent}}_{R}{]}^{2}$+ 2.4[${\mathrm{Ent}}_{R}{]}^{3}$	27.5	32.27
MR	MR= 47.3+4.6[Ent_*ISI*_*]*-16.4[${\mathrm{Ent}}_{ISI}{]}^{2}$+6.3[${\mathrm{Ent}}_{ISI}{]}^{3}$	69.5	84.67
MR	MR= 46.6+3.7[Ent_*R*_*]*-16.3[${\mathrm{Ent}}_{R}{]}^{2}$+6.2[${\mathrm{Ent}}_{R}{]}^{3}$	69.5	80.18

**Table 16:** Comparison between experimental and calculated values of Larotrectinib.

Properties	Regression models	Experimental	Calculated
		values	values
MW	MW= 323.0 +18.4[Ent_*ISI*_*]*- 75.9[${\mathrm{Ent}}_{ISI}{]}^{2}$+24.7[${\mathrm{Ent}}_{ISI}{]}^{3}$	428.4	531.9
C	C=756.7+4.4[Ent_*F*_*]*-309.8[${\mathrm{Ent}}_{F}{]}^{2}$+88.3[${\mathrm{Ent}}_{F}{]}^{3}$	659	788.5
C	C = 724.6+2.1[Ent_*R*_*]*-295.3[${\mathrm{Ent}}_{R}{]}^{2}$+84.4[${\mathrm{Ent}}_{R}{]}^{3}$	659	775.59
P	P = 13.8+1.7[Ent_*R*_*]*-5.8[${\mathrm{Ent}}_{R}{]}^{2}$+ 2.4[${\mathrm{Ent}}_{R}{]}^{3}$	41.61	53.60
MR	MR= 47.3+4.6[Ent_*ISI*_*]*-16.4[${\mathrm{Ent}}_{ISI}{]}^{2}$+6.3[${\mathrm{Ent}}_{ISI}{]}^{3}$	122.96	137.59
MR	MR= 46.6+3.7[Ent_*R*_*]*-16.3[${\mathrm{Ent}}_{R}{]}^{2}$+6.2[${\mathrm{Ent}}_{R}{]}^{3}$	122.96	130.48

## Integration of machine learning in QSPR analysis

In recent years, the application of machine learning (ML) techniques in chemoinformatics has shown significant promise in enhancing the predictive accuracy of QSAR/QSPR models. These advanced computational methods are particularly adept at capturing complex, non-linear relationships that traditional regression models might overlook. In this study, we propose the integration of ML approach to further refine our QSPR analysis of colorectal cancer drugs. We suggest employing supervised learning algorithms such as Random Forest (RF), Support Vector Machines (SVM), and Gradient Boosting Machines (GBM) to predict the biological activity and physical properties of colorectal cancer drugs based on their topological indices [28]–[31].

These algorithms can handle high-dimensional data and are capable of identifying intricate patterns that may not be evident through conventional regression analysis. Random Forests (RF) are proposed for the identification of dominant indices like *S*(*G*) and *H*(*G*) that collectively govern properties, such as molar refractivity. This is because RF has a strong ability to aggregate weaker predictors (lesser-known entropy indices etc) into robust ensembles when at the same time it maintains ranking feature importance through permutation matrices. In addition to this, Gradient Boosting Machines (GBM) complement this through the mechanism of iterative refinement in predictions via error correction and capturing non-linear relationships critical for properties like logP, where cubic regression exhibits poor correlations. Support Vector Machines (SVM) have their unique strengths in dealing with non-linear relationships and high-dimensional data as we know that these are major challenges in QSPR studies involving multi factorial topological indices.

### Implementation strategy

The implementation of the ML techniques involve the following steps:

1. Data Preprocessing: Normalization and standardization of the dataset to ensure compatibility with M algorithms.
2. Feature Selection: Identification of the most relevant topological indices and molecular descriptors using techniques such as Principal Component Analysis (PCA) or Recursive Feature Elimination (RFE).
3. Model Training: Training of ML models on a subset of the data, with cross-validation to prevent overfitting.
4. Model Evaluation: Assessment of model performance using metrics such as Mean Squared Error (MSE), Root Mean Squared Error (RMSE), and R-squared values.
5. Model Optimization: Hyperparameter tuning and model refinement to achieve optimal predictive performance.

## Conclusion

In this article, we studied the physio-chemical properties of fourteen Colorectal Cancer drugs using ten degree-based entropy indices. We have seen from above statistical data that the molecular weight, complexity, molar volume, molar refractivity and polarizability has strongest correlation with all of the entropy indices. Molecular weight, complexity, molar volume, polarizability and molar refractivity has strong and positive linear relationship with entropy indices as corresponding values lies between 0.74 and 1.0 for all the regression models, but if we compare all the models, in case of cubic regression model, all these properties has strong and positive relationship with all the entropy indices. Topological surface area and Density have weak relationship with all the entropy indices as *r*-values lies between 0.4 and 0.54 which are very small. Molecular weight, complexity, molar volume, polarizability and molar refractivity can be predicted using Ent_*ABC*_, Ent_*F*_, Ent_*GA*_, Ent_*H*_, Ent_*ISI*_, ${\mathrm{Ent}}_{M_{1}}$, ${\mathrm{Ent}}_{M_{2}}$, Ent_*S*_, Ent_*SO*_ or Ent_*R*_, as corresponding values of coefficient of determination is high showing that these are the best predictors and good fit models.

Across all the models, cubic regression model is the best predictive model, for molecular weight, complexity, molar volume, polarizability and molar refractivity. But, quadratic models can also considered predictive model particularly for polarizability and molar refractivity. The *p*-value for all the properties is less than or equal to 0.05 except for TSA and density. Thus, all models are best fit for all the properties except for TSA and density. We have seen that in QSPR analysis, degree-based entropies are highly useful. The correlation coefficients of the model under consideration plays a significant role to explore the characteristics of innovative drugs. Moreover, the integration of ML techniques is expected to enhance the predictive accuracy of our QSPR models, providing a more robust framework for the design and optimization of colorectal cancer drugs. This approach will not only improve our understanding of the molecular properties that influence drug efficacy but also pave the way for the development of more effective therapeutic agents. By incorporating these advanced computational methods, we aim to set a new standard in the field of chemo-informatics, demonstrating the potential of AI/ML/DL in revolutionizing drug discovery and development processes. Results obtained in this work will be helpful for the researchers or chemists working in pharmaceutical fields for the further development of drugs and exploring their physio-chemical properties.
